# Pyridinium Rotor Strategy toward a Robust Photothermal Agent for STING Activation and Multimodal Image-Guided Immunotherapy for Triple-Negative Breast Cancer

**DOI:** 10.1021/jacs.4c15534

**Published:** 2025-02-20

**Authors:** Shipeng Ning, Ping Shangguan, Xinyan Zhu, Xinwen Ou, Kaiyuan Wang, Meng Suo, Hanchen Shen, Xiuxin Lu, Xianqing Wei, Tianfu Zhang, Xiaoyuan Chen, Ben Zhong Tang

**Affiliations:** ‡ Department of Breast Surgery, The Second Affiliated Hospital of Guangxi Medical University, Nanning 530000, China; § Guangzhou Institute of Cancer Research, the Affiliated Cancer Hospital, School of Biomedical Engineering, Guangzhou Medical University, Guangdong 511436, China; ∥ Department of Chemistry, Hong Kong Branch of Chinese National Engineering Research Center for Tissue Restoration and Reconstruction, Division of Life Science, and State Key Laboratory of Molecular Neuroscience, The Hong Kong University of Science and Technology, Kowloon, Hong Kong, China; ⊥ Department of Pharmaceutics, Wuya College of Innovation, Shenyang Pharmaceutical University, Shenyang, Liaoning 110016, P. R. China; # Departments of Diagnostic Radiology, Surgery, Chemical and Biomolecular Engineering, and Biomedical Engineering, Yong Loo Lin School of Medicine and Faculty of Engineering, National University of Singapore, Singapore 119074, Singapore; ∇ Clinical Imaging Research Centre, Centre for Translational Medicine, Yong Loo Lin School of Medicine, National University of Singapore, Singapore 117599, Singapore; ○ Nanomedicine Translational Research Program, Yong Loo Lin School of Medicine, National University of Singapore, Singapore 117597, Singapore; ◆ Institute of Molecular and Cell Biology, Agency for Science, Technology, and Research (A*STAR), 61 Biopolis Drive, Proteos, Singapore 138673, Singapore; ¶ School of Science and Engineering, Shenzhen Institute of Aggregate Science and Technology, The Chinese University of Hong Kong, Shenzhen (CUHK-Shenzhen), Guangdong 518172, China

## Abstract

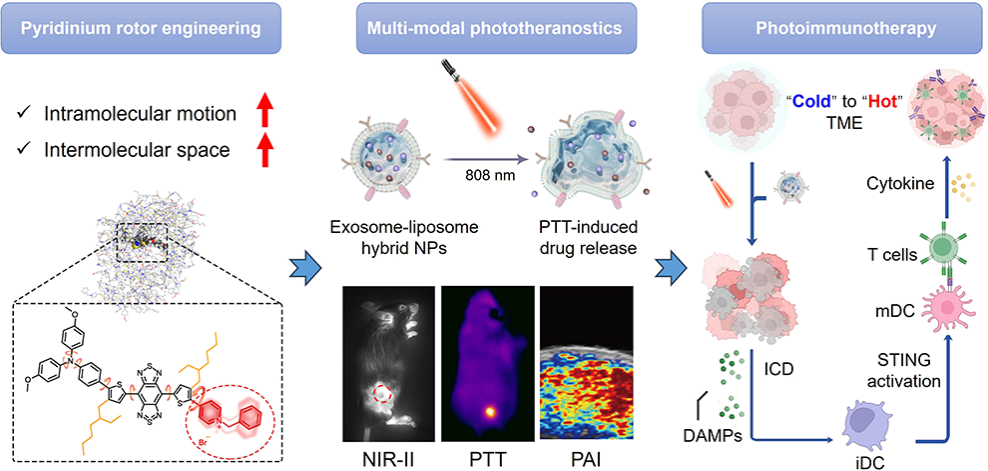

The immunosuppressive tumor microenvironment in triple-negative breast cancer could hinder the response to thorough immunotherapy and diminish the antitumor efficacy. Although the STING pathway emerges as a promising target to remedy defects, uncertain drug delivery might lead to off-target inflammatory reactions. Here, we manifest a novel phototheranostic agent with an aggregation-induced emission property that guided the pharmacological activation of a STING agonist for photothermal immunotherapy to create an immunologically “hot” tumor. A pyridinium rotor strategy is proposed to develop a positively charged TBTP-Bz, which is stably coincorporated with a STING agonist MSA-2 into thermal-responsive exosome-liposome hybrid nanoparticles for tumor-targeting delivery. TBTP-Bz exhibits aggregation-enhanced NIR-II emission and a photoacoustic signal, accomplishing real-time tumor tracking. Its photothermal stimulation induces immunogenic cancer cell death and promotes the precise release of MSA-2, thus boosting STING activation and STING-mediated type I interferon production. Significantly, single-dose photoimmunotherapy effectively suppresses abscopal tumor growth and provokes an immune memory effect to inhibit postsurgical recurrent and rechallenged tumors. This demonstrates promising clinical potential for poorly immunogenic breast cancer.

## Introduction

Despite continuing improvement in clinical treatment modalities including radiotherapy,^1^ chemotherapy,^2,3^ and surgery^4^ to initially eliminate most breast cancers, patients with aggressive subtypes like triple-negative breast cancer (TNBC) often experience high rates of recurrence and metastasis, posing a challenging issue of cancer-induced mortality.^5^ In recent years, cancer immunotherapy utilizing drugs to activate the endogenous immune system, such as inducing immunogenic cancer cell death (ICD) to elicit antitumor effect, has demonstrated outstanding clinical efficacy and sustained therapeutic outcomes across various cancer types.^6−10^ However, TNBC could orchestrate immunosuppression in the tumor microenvironment (TME), leading to immune evasion and weak therapeutic response.^11−13^ Currently, immunotherapy for immunologically cold tumors has been challenging. Therefore, the key lies in selectively stimulating the immune system while effectively preventing immune escape.^14^

The stimulator of interferon genes (STING) is an intracellular signaling receptor that emerges as a critical pathway for governing the innate antitumor immune response.^15^ STING pathway activation can induce type I interferon (IFN) secretion and proinflammatory cytokine expression to remodel the immunosuppressed TME.^16−19^ It has been documented that triggering STING in cancer cells and specific immune cells such as dendritic cells (DCs) can enhance the ability of DC to cross-present antigens to CD8^+^ T cells, which induces spontaneous tumor-specific T cell production and boosts antitumor effects.^20,21^ However, the majority of developed cyclic dinucleotide-based drugs require intratumoral injection, limiting their efficacy and potentially leading to uncontrolled systemic spread and widespread inflammatory reactions.^22,23^ MSA-2 is a newly developed non-nucleotide STING agonist.^24^ While this agent has been administered orally and intravenously in animal studies, its low bioavailability and insufficient cytosolic entry may constrain its antitumor effectiveness. Furthermore, reports suggest that excessive STING activation in immune cells may result in T cell death, promoting cancer cell survival and reinforcing an immunosuppressive environment.^17,25,26^ Therefore, precise and gentle stimulation of the STING pathway in targeted cells is crucial for this therapy.

Photothermal therapy (PTT), which sensitizes and kills cancer cells by heat, has demonstrated the ability to activate inflammation and enhance the efficacy of immunotherapy, garnering significant attention.^27−31^ Organic fluorophore-based photothermal agents have been extensively developed due to their promise for imaging-guided therapies. Yet, the planar structures of traditional fluorescent materials often result in π–π stacking conformation and insufficient electron transition in physiological environments, significantly compromising their luminescence and photothermal conversion efficiency.^32,33^ Of note, the photothermal agent with aggregation-induced emission (AIE) properties and intense fluorescence in the second near-infrared region (NIR-II, 1000–1700 nm), as an advanced conceptual visual theranostic platform, can effectively avoid molecular stacking by incorporating molecules with rotors and twisted structures.^34−36^ Controlled molecular rotation in their aggregated state enables the precise regulation of fluorescent intensity and photothermal efficiency.

**Figure 1:**
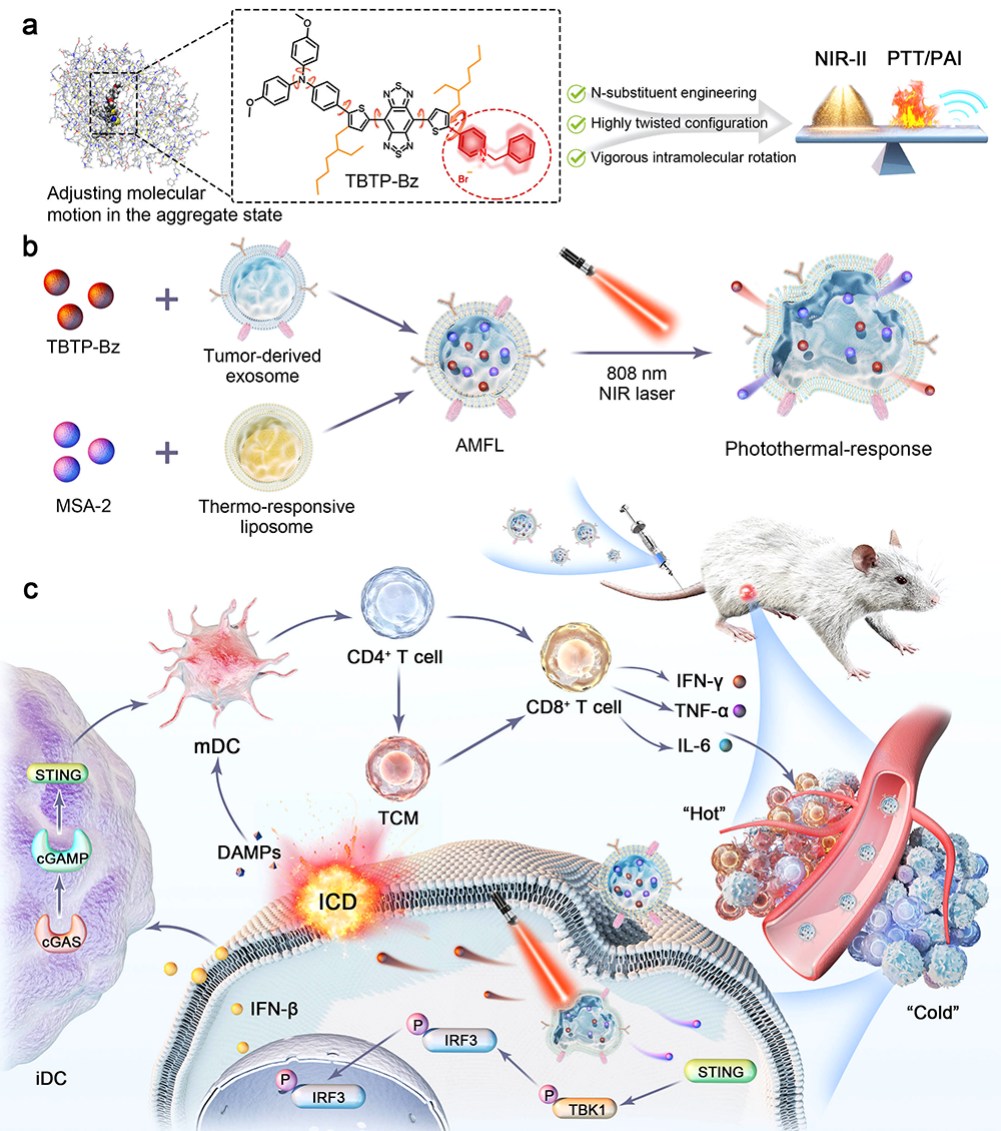
Schematic diagram. (a) Rational design of the AIE NIR-II emissive photothermal agent by adjusting molecular motion in the aggregate state. (b) Codelivering of the AIE phototheranostic agent, TBTP-Bz, and the STING agonist, MSA-2, by exosome–liposome hybrid AMFL. (c) Tumor-targeting AMFL with thermoresponsiveness facilitates drug delivery for synergistic photothermal immunotherapy of breast cancer via STING pathway activation.

**Figure 2:**
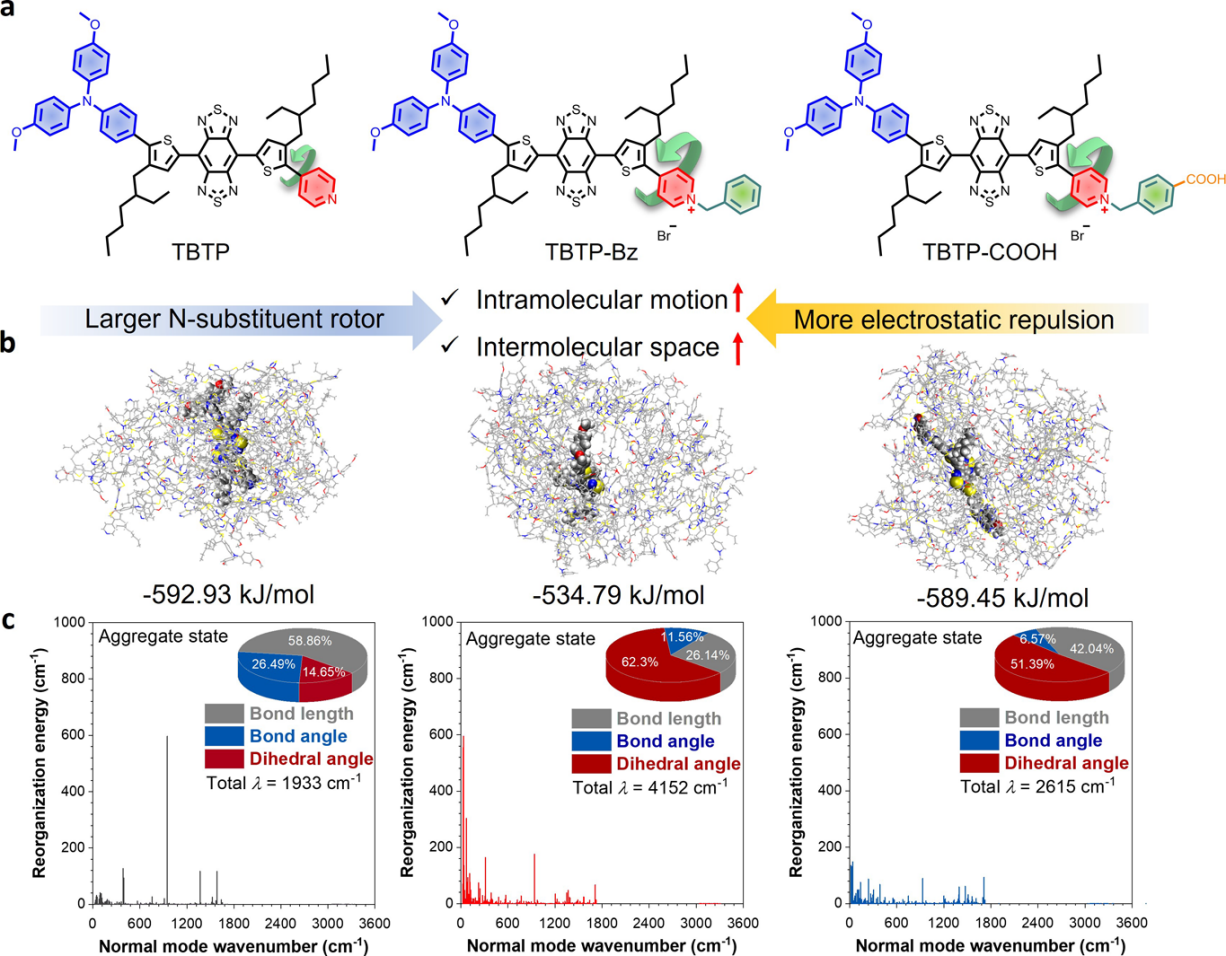
Optimization of AIE phototheranostic agents and their theoretical investigation. (a) Molecular structures of TBTP, TBTP-Bz, and TBTP-COOH. (b) Molecular dynamic simulation models of TBTP, TBTP-Bz, and TBTP-COOH aggregate in water. (c) Plots of reorganization energy vs normal mode wavenumber of TBTP, TBTP-Bz, and TBTP-COOH in the aggregate state. Inset: The proportions of bond length, bond angle, and dihedral angle contributed to the total reorganization energy.

Based on the above-mentioned considerations, we aim to design highly efficient AIE phototheranostic agents that could enhance STING activation through molecular modulation. We innovatively propose a pyridinium rotor strategy, where the optimized molecular rotor is the N-substituent group of the pyridinium structure. This strategy can simultaneously enhance molecular rotation and intermolecular charge transfer, thus balancing the energy consumption pathway in the aggregated state. The optimized TBTP-Bz molecule exhibits efficient photothermal conversion efficiency and aggregation-enhanced NIR-II emission under 808 nm laser irradiation, enabling NIR-II fluorescence imaging (FLI)/photoacoustic imaging (PAI)/photothermal imaging (PTI)-guided PTT (Figures 1a and 2a). At the same time, the precision of molecular targeting is crucial to ensure efficient local heat-induced cancer cell ablation while minimizing damage to normal tissues. Furthermore, enhancing the internalization efficiency of nanomaterials plays a significant role in promoting photothermal immunotherapy, which enhances STING activation and antigen presentation.^37,38^ In recent studies, researchers have utilized tumor-derived exosomes to encapsulate or fuse with nanomaterials, increasing the tumor-targeting ability and cellular internalization rate of nanomedicine through surface topological modifications.^39−41^

Inspired by the intelligent process, a thermoresponsive biomimetic nanoparticle is fabricated for precise drug delivery and selectively leveraging innate antitumor immunity under NIR laser stimulation. Specifically, we fused the temperature-sensitive liposomal 1,2-dipalmitoyl-*sn*-glycero-3-phosphorylcholine (DPPC) and 4T1 cancer cell-derived exosomes that entrap TBTP-Bz and MSA-2, forming what is termed AMFL (Figure 1b). Upon accumulation and infiltration into the tumor region facilitated by abundant tumor receptors on the exosome surface, the AIE phototheranostic agent exhibits intense NIR-II fluorescence, photoacoustic and photothermal signals with high signal-to-noise ratio, enabling real-time tracking of the tumor site in the three modalities. The photothermal effect based on the AIE phototheranostic agent promotes the deformation of DPPC, leading to a controlled release of MSA-2 for enhancing STING pathway activation in cancer cells. Additionally, the ICD induced by the AIE phototheranostic agent effectively triggers the release of damage-associated molecular patterns, demonstrating a synergistic enhancement with STING agonists, involving downstream immunostimulatory signal production and IFN secretion. Further investigation reveals that the STING signaling in DCs was cooperatively activated to elicit DC maturation and anticancer T cell priming for robust adaptive immune responses (Figure 1c). Consequently, TNBC suppression, potent inhibition of distant tumors, and effective resistance to postsurgical recurrent and rechallenged tumors are clearly demonstrated in mice models. Hence, this study highlights the potential of AIE-based PTT augmenting STING agonist activation for the synergistic photothermal immunotherapy of TNBC.

## Results and Discussion

### Facilitation of Molecular Motion to Develop NIR-II Emissive AIE Photothermal Agents

A series of molecules with D−π–A-type structures were designed consisting of three compositions: the methoxy triphenylamine and pyridine moiety served as the electron donor (D) and the electron acceptor (A), respectively. A low-bandgap benzo[1,2-*c*:4,5-*c*′]bis([1,2,5]thiadiazole) acceptor and a π-conjugated thiophene ring with bulky alkyl chains were strategically integrated as the extended π-bridge and intermolecular spacer. This design aimed to fortify the D–A interaction and facilitate intramolecular charge transfer, thereby reducing the electronic bandgap and extending the wavelengths of absorption and emission. In addition, the twisted conformation of triphenylamine and the incorporated alkyl chains effectively deter intermolecular π–π stacking, fostering a pronounced AIE effect and heightened fluorescence in the aggregate state. A benzyl group was added as a N-substituent of the pyridinium moiety, creating an additional intermolecular space and enhancing intramolecular motion. Consequently, TBTP-Bz with positive charges was synthesized. TBTP and TBTP-COOH were synthesized as controls (Figure 2a). All of the molecules were synthesized in good yields, ranging from 75 to 86% (Scheme S1), and their structures were fully characterized by NMR and high-resolution mass spectroscopies (Figures S1–S6). The optical properties of TBTP, TBTP-Bz, and TBTP-COOH were investigated. The absorption spectrum of the molecules peaked at 805–835 nm in the aggregate state (Figure S7). The photoluminescence (PL) signals were detected within the 900–950 nm range, with a broad band spanning the NIR-II region 1000–1550 nm. With NIR-I absorption and NIR-II fluorescence, these molecules proved ideal for deep tissue imaging with a high signal-to-noise ratio. The AIE characteristics of TBTP, TBTP-Bz, and TBTP-COOH were validated in different good/poor solvent mixtures. As depicted, TBTP, TBTP-Bz, and TBTP-COOH exhibited weak emissions in THF or DMF, yet showed intensified PL signals in 90 or 99% of the poor solvent conditions (Figure S8). To preliminarily assess their photothermal potential in physiological settings, an 808 nm laser was leveraged to align with their absorption peaks with enhanced tissue penetration depth. Experiments demonstrated that TBTP-Bz could attain temperatures of up to 72.9 °C after 6 min of laser irradiation with a concentration-dependent and laser power-dependent effect (Figure S9a–c). Notably, TBTP-Bz exhibited the highest photothermal conversion efficiency of up to 58.5% among the three AIE molecules (Figure S9). Furthermore, the photothermal stability and resistance to photobleaching of TBTP-Bz aggregates were confirmed through consistent temperature maintenance over three cycles of on–off heating. The high photothermal conversion efficiency of TBTP-Bz in its aggregate state prompted further investigation into its underlying mechanisms using molecular dynamics (MD) simulations with the GROMACS 2020 package. To obtain amorphous aggregates of the AIE molecules in physiological environments, 40 molecules were randomly placed in a cubic box for initial aggregation and then mixed with surrounding water molecules (Figure S10a–c). Energy minimization using the steepest descent algorithm was performed to relax the system. The optimized snapshots of aggregates (Figure 2b) were picked from the production simulation to show the packing manner of the AIE molecules in the aggregate state, and the innermost molecules were chosen to study the stacking mode. TBTP-Bz exhibited the weakest intermolecular interaction (−534.79 kJ/mol) compared to the others (−592.93 and −589.45 kJ/mol). Among all kinds of interactions, TBTP-Bz has a weak electrostatic interaction that could be attributed to the electronic repulsion of the positively charged pyridinium moiety. In comparison, the −COOH group in TBTP-COOH counteracts some of the repulsion effects from the positively charged group, showing a smaller intermolecular space and larger van der Waals interactions. Figure S10d–f depicts that TBTP-Bz exhibited a loose packing as it shows a smaller atomic contact ratio (84.9%) compared to the other molecules (92.0% for TBTP and 87.3% for TBTP-COOH). Similarly, the optimized ground-state and excited-state geometries of TBTP-Bz calculated by the density functional theory method showed an obvious dipole electrostatic distribution (Figure S11a–c).

TBTP-Bz displayed the highest zeta potential (62.0 mV) among three molecules from dynamic light scattering (DLS) (Figure S12), testifying to the results from MD that the positively charged moiety of TBTP-Bz plays an important role in its packing mode. Next, the single-molecule and aggregated reorganization energies (λ), a quantitative analytical parameter of intrinsic geometry change upon photoexcitation representing the contribution of intramolecular motion, were calculated to evaluate the energy consumption from the excited state to the ground state through nonradiative pathways (Figure S11). TBTP-Bz single molecules and aggregates showed large total λ values of 12,922 and 4152 cm^–1^, respectively, with a high contribution of dihedral angle associated with molecular motions (81.6% for the gas phase and 62.3% for the aggregate state). The results from the calculation were in accordance with the strong photothermal conversion efficiency of TBTP-Bz from the experimental results. The nonradiative decay of the excited state of TBTP-Bz can be triggered by the drastic molecular motion of the large-sized and the electrostatic repulsion of the positively charged pyridinium moiety, whereas these interactions could significantly lose the molecular packing and promote the balance of the radiative and nonradiative decay (Figures 2c and S11c).

### Fabrication and Characterization of a Hybrid Biomimetic Liposomal System

**Figure 3:**
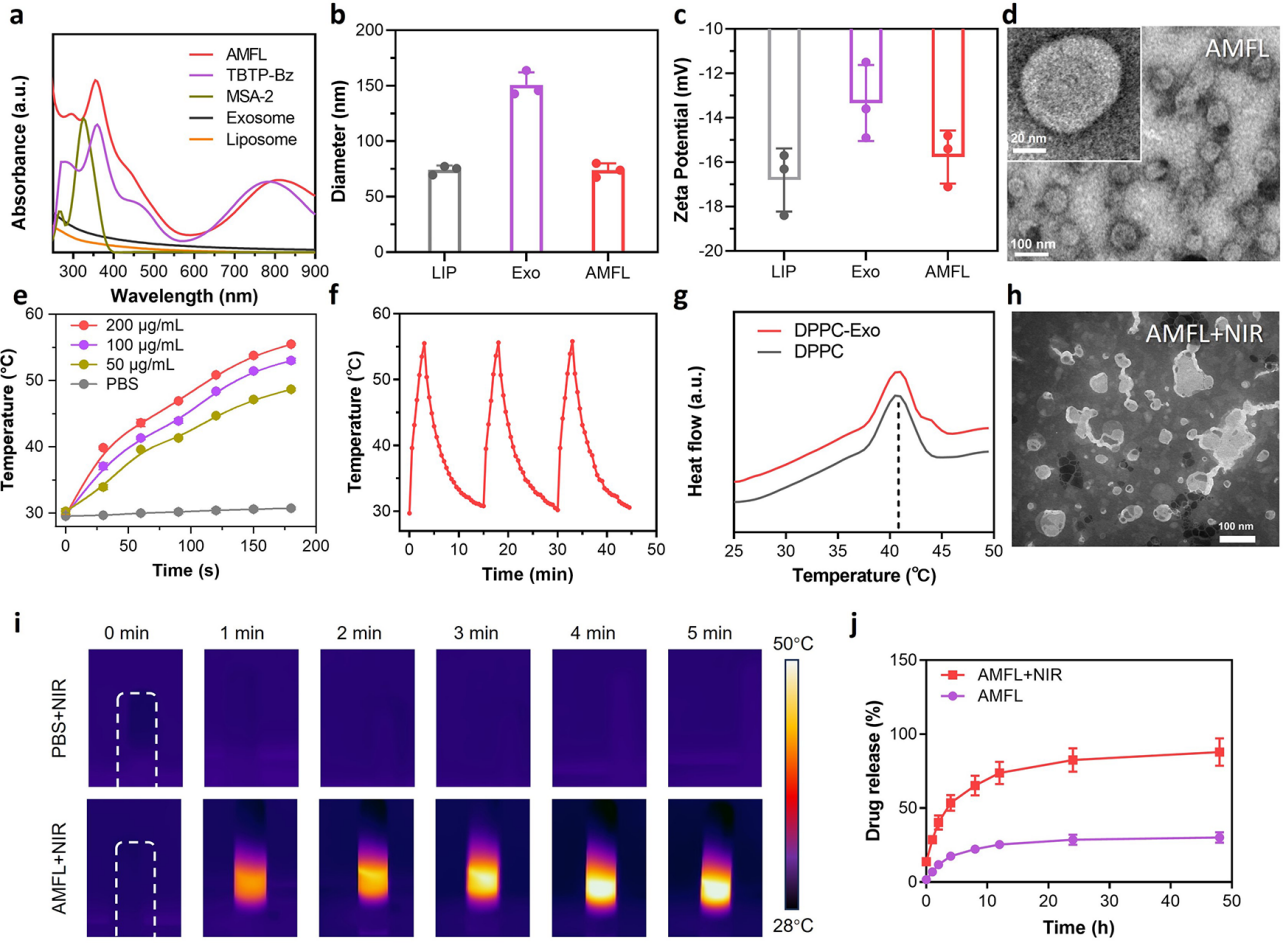
Fabrication and characterization of AMFL. (a) Absorption spectrum of AMFL, TBTP-Bz, MSA-2, liposome, and 4T1 cell-derived exosome membrane. (b and c) DLS analysis on the size distribution and zeta potential of the pure DPPC liposome, exosome, and AMFL. (d) TEM images of AMFL for illustrating the morphological details. (e) Temperature change curves of AMFL with different concentrations of TBTP-Bz under NIR laser exposure (808 nm, 0.5 W/cm^2^). (f) Photothermal stability of AMFL loaded with 200 μg/mL TBTP-Bz in water during three cycles of heating–cooling processes. (g) Experimental heat flow of liposomal DPPC and exosome–liposome hybrid nanoparticles measured by the DSC method. (h) TEM images of AMFL after NIR laser exposure (808 nm, 0.5 W/cm^2^, 5 min). (i) Thermal imaging of the PBS and AMFL solution after NIR laser exposure (808 nm, 0.5 W/cm^2^). (j) Time-dependent release of MSA-2 from AMFL with or without NIR laser exposure (808 nm, 0.5 W/cm^2^) (*n* = 3).

To ensure drug delivery efficiency and therapeutic effect, nanoparticles with balanced tumor-targeting specificity, stimulus-responsive function, and biological barrier penetration ability are desirable. Herein, an exosome-liposome fusion strategy was used to encapsulate TBTP-Bz and MSA-2, obtaining AMFL. The exosome–liposome hybrid nanoparticles (FL) were fabricated via the thin-film hydration method. The absorption and FTIR spectrum of AMFL exhibited characteristic peaks corresponding to TBTP-Bz and MSA-2, confirming the successful loading of both molecules (Figures 3a and S13). Western blot (WB) analysis revealed the presence of CD9 and CD63 proteins in AMFL and exosome membranes (Figure S14). Since CD9 and CD63 are characteristic surface proteins found on 4T1 cell-derived exosomes, their expression in AMFL indicates the successful integration of exosome membrane components, imparting it with tumor-targeting capability. DLS measurements demonstrated that the size and zeta potential of AMFL remained consistent with those of liposomes, ensuring the stability of the hybrid nanoparticles (Figures 3b,c and S15). Transmission electron microscopy (TEM) imaging of AMFL revealed spherical vesicles with a diameter of ∼80 nm (Figures 3d and S16). As depicted in Figure 3g, differential scanning calorimetry (DSC) analyses of DPPC and DPPC-exosome fusion nanoparticles exhibited a distinct phase transition process with a similar transition temperature of ∼41 °C, indicative of a cooperative and reversible thermal-responsive gel-to-fluid transition. Subsequently, the photothermal properties of AMFL and its capability for controlled drug release were evaluated (Figures 3e–h and S17). Upon exposure to 808 nm laser irradiation, AMFL nanoparticles in TEM images showed an expanded and disintegrated morphology, which could be attributed to the thermal-responsive nature of the liposomal component under the photothermal effects of TBTP-Bz (Figure 3h). In addition, the efficient release of MSA-2 was realized under the photothermal effect of AMFL (Figure 3i,j). Furthermore, confocal microscopy images demonstrated that AMFL exhibited enhanced internalization by cancer cells compared to AML, which contained only liposomes as encapsulated materials, underscoring its potent and cancer cell penetration ability (Figure S18).

### In Vitro Anticancer Power with Enhanced STING Pathway Activation

**Figure 4:**
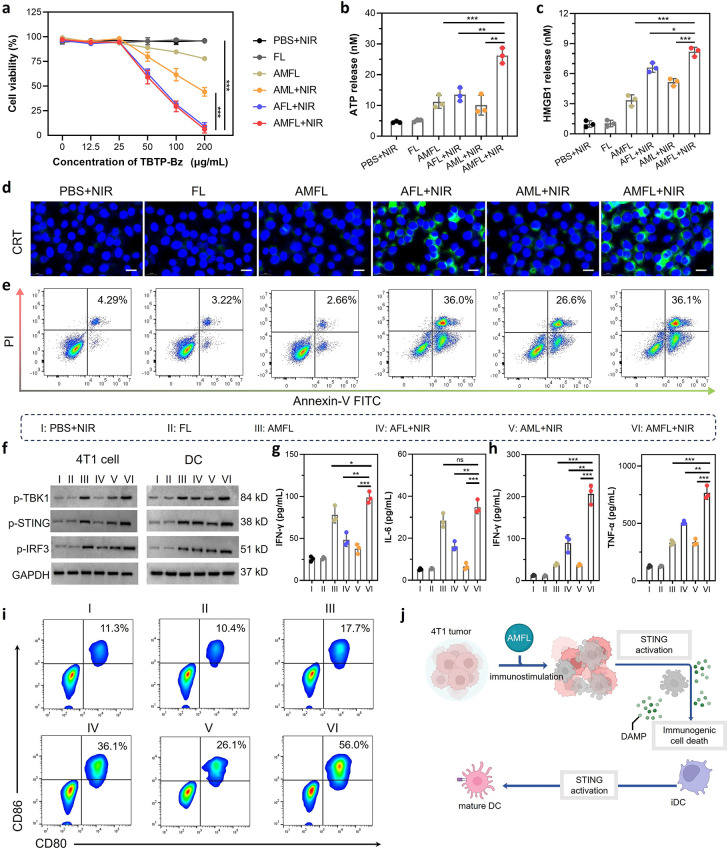
AMFL-induced ICD and selective STING stimulation in DC primes immune response. (a) Cell cytotoxicity after different treatments was determined based on the TBTP-Bz concentration in the AMFL, *n* = 3. (b and c) ATP and HMGB1 levels by ELISA in cell culture supernatants after different treatments, *n* = 3. (d) CLSM imaging of CRT expression after different treatments. Scale bar is 50 μm. (e) Apoptosis ratio after different treatments analyzed by Annexin V/PI assay, *n* = 3. (f) WB analysis of p-TBK1, p-STING, and p-IRF3 in 4T1 cancer cells and DC. (g) Concentration of IFN-γ and IL-6 in 4T1 cancer cells after different treatments. (h) Concentration of IFN-γ and TNF-α in DC after different treatments, *n* = 3. (i) Flow cytometric analysis of the expression levels of DC maturation (DC80/DC86). (j) Illustration of AMFL-stimulated ICD of tumor cells and STING signaling of DCs in the TME. Data are given as means ± SD. Statistical significance was calculated by one-way ANOVA with Tukey’s post hoc test. *P < 0.05, **P < 0.01, and ****P < 0.0001. ns denotes no significant difference. FL, pure exosome–liposome hybrid nanoparticles without drug loading; AML, liposome nanoparticles loaded with TBTP-Bz and MSA-2; and AFL, exosome–liposome hybrid nanoparticles only loaded with TBTP-Bz.

Leveraging the tumor-targeting capabilities and photothermal-responsive drug release functionality, the anticancer efficacy was assessed. First, preliminary studies were performed to ensure the therapeutic potential of TBTP-Bz over those of the other two molecules. The FL encapsulating TBTP-Bz was designated as AFL, and the FL encapsulating TBTP and TBTP-COOH were designated as AFLa and AFLc, respectively. The Cell Counting Kit-8 (CCK-8) cell viability assay and thermal imaging recording demonstrated that TBTP-Bz has the highest phototoxicity and in vivo photothermal effect in tumor-bearing mice (Figures S19 and S20). Next, the in vitro efficacy of AMFL was evaluated. AFL and the pure liposomes encapsulating both TBTP-Bz and MSA-2 were designated as AML, as control groups. The CCK-8 cell viability assay demonstrated that AMFL exhibited negligible cytotoxicity toward 4T1 cells, consistent with prior findings that MSA-2 does not induce direct cytotoxic effects on cancer cells. AML displayed moderate cytotoxicity under laser irradiation, with a cell viability of 44.1% ± 4.4% even at high concentrations of TBTP-Bz and MSA-2, possibly due to its inadequate cancer cell internalization. Under NIR laser irradiation, AMFL and AFL exhibited significantly enhanced cytotoxicity by killing over 90% of the 4T1 cancer cells, showcasing the photothermal properties of TBTP-Bz and efficient cellular delivery with FL encapsulation (Figures 4a and S21). Subsequently, flow cytometry was employed to evaluate the cell apoptosis ratio by annexin V-FITC/PI staining (Figure 4e). The proportion of apoptotic cells surged to 36.1% in the AMFL-treated groups following laser irradiation, surpassing the control groups and signifying the onset of proapoptotic processes. To elucidate the mechanism behind the AMFL-mediated superior antitumor efficacy, PTT-induced ICD, DC maturation, and relevant immune markers were investigated. The typical characteristics of ICD, such as adenosine triphosphate (ATP) release, calreticulin (CRT) translocation to the cell surface, and high mobility group box 1 protein (HMGB1) expression, were first examined (Figures 4b–d and S22b). These features facilitate antigen uptake, processing, and presentation by DCs, promoting cytotoxic T cell production. As shown in the immunofluorescence staining images, intense fluorescence signals of HMGB1 and CRT were detected in the AFL and AMFL-treated groups under NIR laser irradiation. The level of ATP in the tumors of the AMFL + NIR group exhibited a 5.7-fold increase compared to PBS-treated controls, indicative of ICD induction. After verifying the ICD-evoking ability of AMFL, DC maturation was investigated (Figures S22c and 4i). We treated 4T1 breast cancer cells in different groups and then indirectly cocultured with murine bone marrow-derived dendritic cells (BMDCs) via a Transwell system that allows the transfer of hybrid nanoparticles to the BMDC. The populations of matured DCs increased to 36.1 and 56.0% for AFL + NIR and AMFL+NIR groups, respectively. In contrast, no significant changes in matured DC populations (17.7%) were noted in the AMFL group in the absence of laser irradiation, indicating the limited impact of AMFL on inducing DC maturation without an effective drug release. While ICD induction has been reported to be a potentially promising pathway for immunotherapy, immune escape poses a challenge to its efficacy. To address this, MSA-2 in AMFL serves as the STING-pathway activator to boost the antitumor immune response. To further explore whether AMFL could activate the STING pathway in cancer cells, the related signaling pathway was analyzed. WB analysis confirmed heightened levels of phosphorylated STING, TANK-binding kinase 1 (TBK1), and interferon regulatory factor 3 in AMFL and AMFL + NIR groups compared to control groups that lack MSA-2 (AFL + NIR group), indicating STING pathway activation (Figures 4f and S22). Notably, the STING pathways were stimulated in DCs. We extracted the supernatant of AMFL-treated 4T1 tumor cells post low-speed centrifugation and used it for BMDC conditioning. WB results exhibited elevated expression levels of key STING signaling components following AMFL treatment, notably higher than the other groups (Figure 4f). On the other hand, the efficient DC uptake of AMFL was testified, providing the support that MSA-2 could deliver to DC for STING activation (Figure S23). Additionally, the STING signaling components in the AMFL+NIR group promoted IFN-β and TNF-α production in 4T1 cells and DCs, along with increased levels of proinflammatory cytokines IFN-γ and IL-6 (Figure 4g,h). This underscores AMFL’s ability to dual-stimulate ICD and STING pathways, thus enhancing DC maturation and its antigen-presenting power (Figure 4j).

### Real-Time In Vivo Tumor Diagnosis via Multimodality Imaging

**Figure 5:**
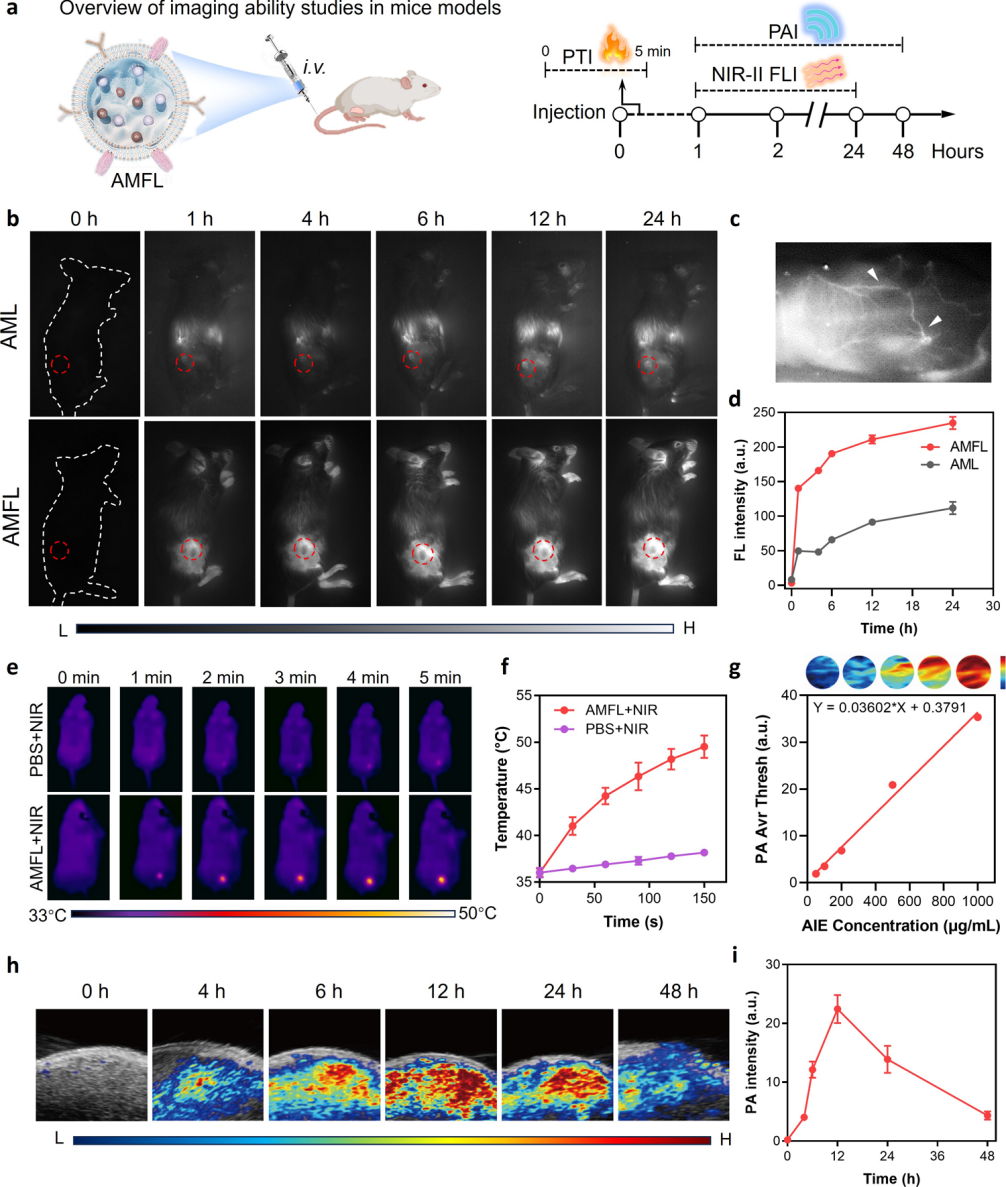
In vivo multimodel tumor imaging by AMFL. (a) Schematic illustration showing the experimental process. (b) In vivo real-time NIR-II FLI of tumor (red dashed line) at different postinjection times of AML or AMFL by the 1250 nm long-pass filter. The concentration was 10 mg/kg. The white dashed lines outline the mice position in 0 h, and the red dashed circles outline the tumor sites. (c) NIR imaging of blood vessel section in mice treated with AMFL at 5 min post injection; the white arrow points out the blood vessel position. The concentration was 10 mg/kg. (d) Quantification of the NIR-II fluorescence intensity after treatment with AML and AMFL at different time points. Data are presented as the mean ± SD (*n* = 3). (e) In vivo PTI and (f) the corresponding quantification curve after treatment with PBS or AMFL upon irradiation (808 nm, 0.5 W/cm^2^) in 5 min. Data are presented as mean ± SD (*n* = 3). (g) PA intensity of different concentrations of TBTP-Bz in AMFL. The inset image represents PA images of AMFL solutions with different concentrations of TBTP-Bz. (h) In vivo PA images and (i) the corresponding quantification curve of tumors from AMFL-administrated mice. Data are presented as mean ± SD (*n* = 3). AML, liposome nanoparticles loaded with TBTP-Bz and MSA-2.

To further validate the suitability of AMFL for in vivo theranostics and investigate its potential for clinical application, we assessed the tumor-targeting capability in 4T1-bearing mice using multimodal imaging techniques (Figure 5a). Initially, AMFL solution was imaged to show concentration-dependent NIR-II signals under 808 nm laser excitation (Figure S24). Then it was intravenously administered to the tumor-bearing mice, and tumor accumulation was monitored through NIR-II FLI. As a control, AML that utilizes pure liposomes as encapsulating materials was also injected under the same conditions. The tumor structure was imaged with 1250 nm bypass at different time points. As shown in Figure 5b, a clear resolution of tumor features was observed with gradually increased NIR-II fluorescence intensity in the AMFL groups. The intensity reached the platform at 12 h post injection and remained steady even at 24 h post injection, indicating its long-term retention potential (Figure 5d). Longer imaging wavelengths enhanced the spatial resolution of fine structures of the tumor area by remarkably increasing the signal-to-noise ratio. In comparison, mice treated with AML displayed a much weaker NIR-II fluorescence signal in tumor regions, indicating the effective tumor accumulation of AMFL. Furthermore, in vivo biodistribution also showed similar results that AMFL possessed higher tumor accumulation and retentions (Figures S25 and S26).^42^ Additionally, we intravenously injected AMFL into a mouse’s bloodstream, and its angiography was recorded. The whole vessel network of the mouse is clearly visualized in prone positions, providing more accurate diagnostic information about early diseases (Figure 5c). Next, the in vivo PTT potential of AMFL was investigated (Figure 5e). After intravenous injection of AMFL for 12 h, the tumor temperatures of the mice were monitored over time using IR thermography while being exposed to 808 nm laser irradiation. Figure 5f demonstrates the obvious temperature rise in AMFL-treated mice with a maximum temperature of 50.6 °C upon irradiation (0.5 W/cm^2^) for 5 min, while mice receiving PBS showed negligible temperature elevation in the same condition. Inspired by the promising photothermal effect, the application of AMFL in PA imaging was studied. The direct correlation observed between the PA intensity and the concentration of the AMFL underscores the significant potential in facilitating quantitative PA imaging (Figure 5g). In vivo PA tumor imaging was conducted by intravenous injection of AMFL into 4T1 tumor-bearing mice. Figure 5h depicts the PA images of the tumor at different time points. Intense PA signal was distinctly visible at the tumor site at 4 h post injection and reached its peak around 12 h post injection, mirroring the results obtained from NIR-II imaging (Figure 5i).

### In Vivo Inhibition of TNBC, Suppression of Abscopal Tumor, and Control of Postsurgical Recurrent and Rechallenged Tumors

**Figure 6:**
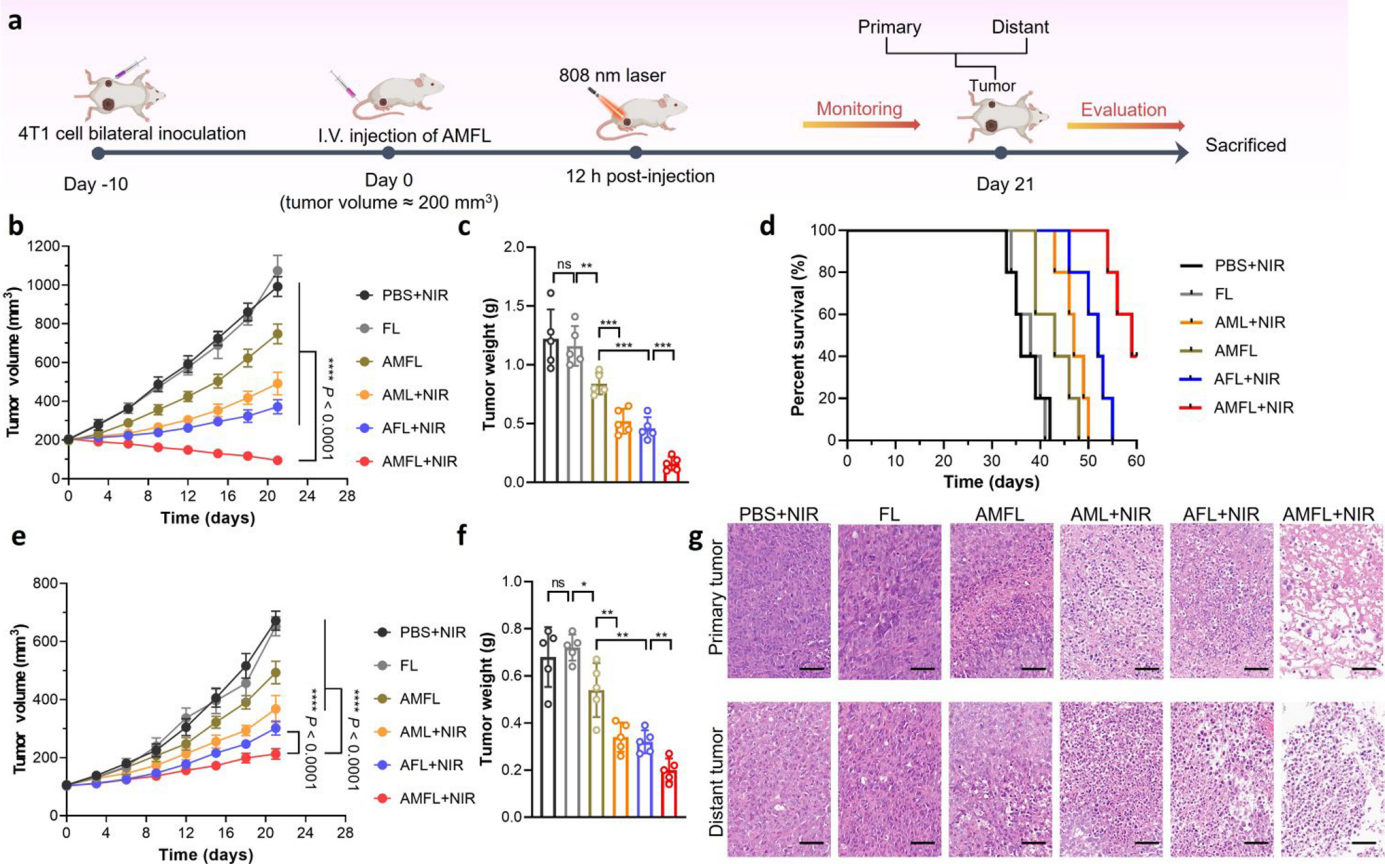
In vivo antitumor therapy by tumor-specific AMFL in bilateral 4T1 tumor-bearing mice. (a) Overview of the experimental design. Treatments were initiated on primary tumors for one injection and irradiation. (b) Tumor volume of the primary tumor over time after injection of different treatments. (c) Tumor weight of primary tumors collected from sacrificed mice after different treatments on day 21. Data are given as mean ± SD (*n* = 5). (d) Survival of mice after different treatments. (e) Tumor volume of the distant tumor over time after injection of different treatments. (f) Tumor weight of distant tumors collected from sacrificed mice after different treatments on day 21. (g) Representative H&E staining images of tumors from indicated groups. Scale bar is 50 μm. Data are given as mean ± SD (*n* = 5). Statistical significance is calculated via one-way ANOVA with Tukey’s test: *p < 0.05; **p < 0.01; ***p < 0.001; ****p < 0.0001, ns denotes no significant difference. FL, pure exosome–liposome hybrid nanoparticles without drug loading; and AML, liposome nanoparticles loaded with TBTP-Bz and MSA-2.

**Figure 7:**
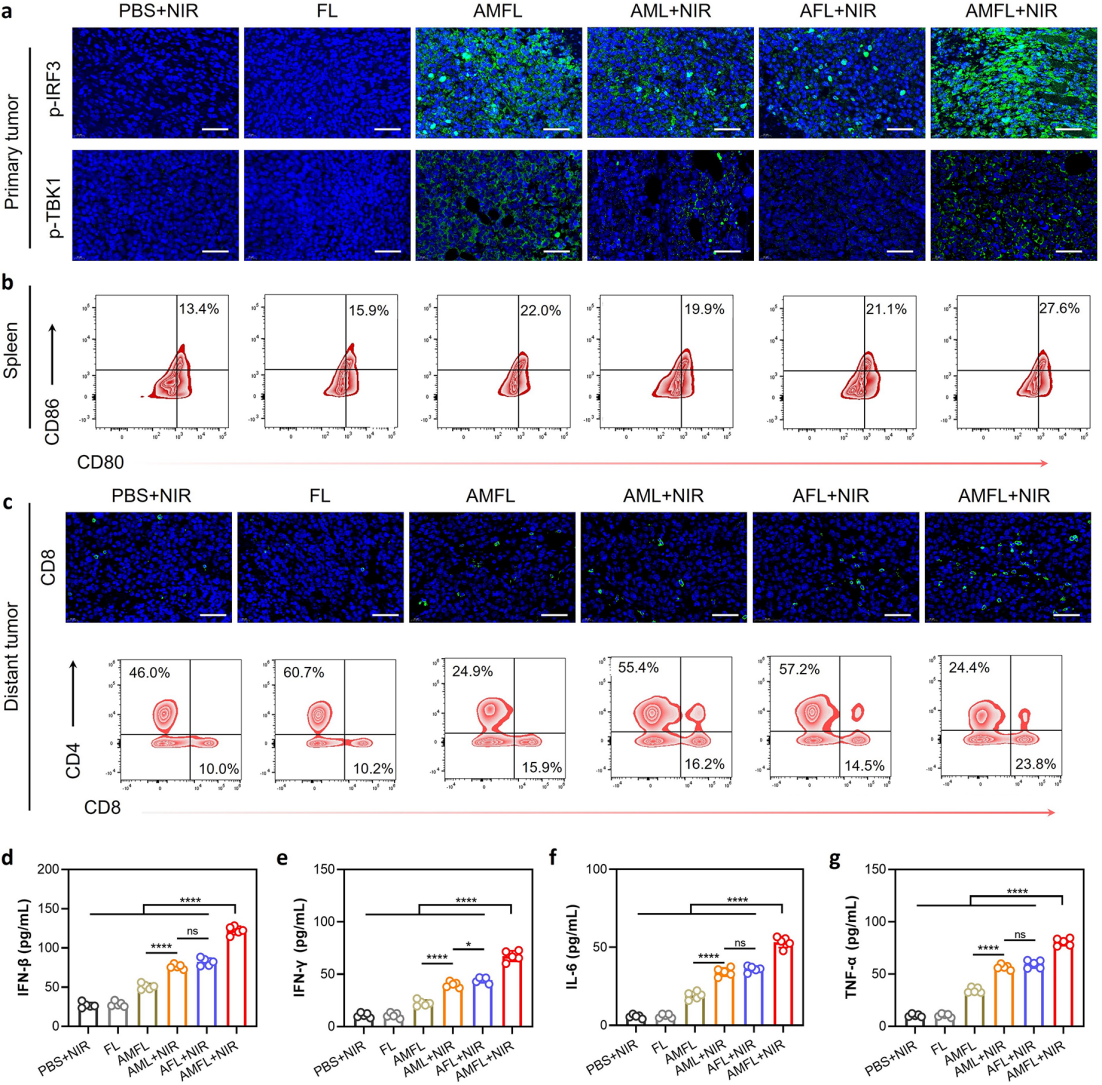
AMFL enhanced antitumor immunity. (a) Immunofluorescent staining images of p-TBK1 and p-IRF3 of the primary tumor tissues excised from the mice in each group. Scale bar is 50 μm. (b) Flow cytometry analysis of DC maturation (CD80/CD86) in the spleen after different treatments. (c) CD8 immunofluorescent staining and flow cytometry analysis of T cell activation (CD4/CD8) in the distant tumor after different treatments. Scale bar is 50 μm. ELISA results of (d) IFN- β, (e) IFN-γ, (f) IL-6, and (g) TNF-α in distant tumor tissues under different treatments. Data are given as mean ± SD (*n* = 5). Statistical significance was calculated via one-way ANOVA with Tukey’s test: *p < 0.05; ****p < 0.001, ns denotes no significant difference.

Posteriorly, we further used a bilateral tumor mouse model to investigate the in vivo therapy potential of AMFL as a photothermal-immunotherapeutic trigger. The bilateral tumor model was established by injection of 4T1 cells into the right and left flanks as the primary tumor and distant tumor on day 0, respectively. Mice were randomly assigned to five groups when the primary tumor reached ∼200 mm^3^: PBS + NIR, FL (unloaded exosome–liposome hybrid nanoparticles), AML + NIR, AFL + NIR, AMFL, and AMFL + NIR. The formulations were intravenously injected, and the primary tumor was directly irradiated (808 nm, 0.5 W/cm^2^, 5 min) at 12 h post injection (Figure 6a). The evaluation results of the tumors demonstrated that the AMFL+NIR treatment exhibited effective suppression of primary tumors (Figures 6b,c and S27a) and growth control of distant tumors with the highest survival rate (Figure 6d–f). The body weight of the mice in each group did not change significantly after administration (Figure S27). Histological examination of tumor slices stained with hematoxylin and eosin revealed the efficient destruction of tumor cells in both primary and distant tumors, indicating an abscopal effect of AMFL (Figure 6g). Furthermore, the immunofluorescence analysis of primary tumor slices showed that the mice in AMFL and AMFL+NIR groups have a high expression on p-TBK1 and p-IRF3, indicating the STING activation due to the precise accumulation of MSA-2 on tumors (Figures 7a and S28a,b). Meanwhile, the secretion of STING-related cytokines such as IFN-β, as well as proinflammatory cytokines involving IFN-γ, TNF-α, and IL-6, were measured by ELISA and RT-qPCR, showing a substantial increase in tumor tissues of the AMFL + NIR group compared to the other groups, supporting the STING pathway activation and the potential to convert the “cold” tumor into a more sensitive “hot” tumor (Figures 7d–g and S29). It is worth noting that the intravenously injected pure MSA-2 drug in mice caused significantly higher secretion levels of TNF-α and IFN-β compared to the same concentration of MSA-2 in AMFL, which may lead to unwanted systemic immunity. This indicates the importance of the precise drug release of AMFL (Figure S30). Encouraged by the in vivo therapeutic effect and STING activation, the elevation of systemic immunity was evaluated. As shown in Figures 7b and S31, the expression of mature DCs (CD80^+^CD86^+^) in the lymph nodes and spleen was enhanced by ∼2.6- and 2-fold after AMFL + NIR treatment in contrast with the PBS + NIR groups (Figure S28d), along with CD8^+^ T cell activation in the lymph nodes (Figure S31). The frequency of tumor-infiltrating CD8^+^ T cells exhibited a remarkable rise in distant tumor tissues in the AMFL + NIR groups, confirming the induction of a robust immune response (Figures 7c and S28c). Thus, the mechanism of the abscopal effect can be revealed. Benefiting from the efficient tumor-targeting ability and accumulation effects facilitated by the exosome-liposome hybrid encapsulation, the PTT effect was presented by TBTP-Bz under NIR laser irradiation. This process assisted the infiltration of MSA-2 and the induction of the STING pathway with cytokine secretion. The synergistic therapeutic effect may contribute to DC maturation and subsequent T cell activation in tumor tissues, thereby eliciting an antitumor immune response.

To fully exploit the long-term protection effect of our photothermal-immunotherapeutic trigger and assess its clinical potential,^43−45^ we established a recurrent and rechallenged tumor model in mice (Figure S32a). Following the treatments mentioned earlier, the primary tumor in the right hind leg of the mice was surgically removed on day 4 and the mice were subsequently rechallenged with secondary 4T1 tumors in the left hind leg. The volumes of the recurrent and rechallenged 4T1 tumors were monitored at certain time intervals. Assessment of tumor volume and weight revealed that AMFL+NIR treatment effectively suppressed the malignant growth of both recurrent and rechallenged tumors compared to the other groups (Figures S32b–h and S33a), accompanied by an increased secretion of pro-proinflammatory cytokines such as TNF-α and IFN-γ, ultimately leading to improved survival rates among tumor-bearing mice (Figure S32i–k). To evaluate the long-term antitumor immune responses, we examined the frequency of central memory T cells (TCM, CD62L^+^CD44^+^) collected from mice treated with different regimens (Figure S32l). The frequency of TCM cells significantly rose in the AMFL + NIR group, with the proportion of TCM cells being approximately 2-fold higher than that in the control groups (Figure S33b). This suggests that the functional TCM generated by the AMFL+NIR treatment could provide substantial long-term protection against tumor recurrence and rechallenge. In addition, the TUNEL and H&E staining of tumor slices revealed extensive cell damage in the post-treatment period, with the most severe damage observed in the AMFL + NIR group (Figure S34).

In contrast, the other five control treatment groups exhibited only partial cell damage in their respective tumor slices. Following the treatments, the body weights of mice across all groups remained stable for 21 days, and no significant morphological changes were observed in the main organs (heart, liver, spleen, lung, and kidney). Blood biochemical analysis revealed excellent blood compatibility and the least liver toxicity, with no discernible differences between the groups, indicating that the formulated AMFL exhibited minimal systemic toxicity during the treatment period (Figures S35–S37). These findings underscore the favorable in vivo biosafety profile of AMFL for cancer immunotherapy.

## Conclusions

To this end, we have first proposed a pyridinium rotor strategy to develop simple D–A-type AIE phototheranostic agents. By functionalizing the N-substituents of pyridinium, the increased molecular rotor in the molecules helps with skeleton distortion and twisting. The resulting drastic intramolecular motions of optimized molecules TBTP-Bz will dissipate parts of the excited-state energy via nonradiative decay, leading to an excellent photothermal conversion efficiency of 58.5%. More importantly, TBTP-Bz exhibits aggregation-induced NIR-II fluorescence for highly sensitive NIR-II FLI-guided multimodal tumor diagnosis and efficient phototherapy, which is suitable for potentiating following cancer immunotherapy.

Here, we constructed thermosensitive cancer-targeting AMFL by coating 4T1 TNBC cell exosome membranes and temperature-responsive liposomal DPPC encapsulated with TBTP-Bz and MSA-2. Equipped with specific receptors for 4T1 tumor, intravenously injected AMFL imaged the tumor area via long-retention NIR-II fluorescent emission and photothermal energy-derived PA signal. Upon the tumor-killing photothermal effect generated by TBTP-Bz under 808 nm laser irradiation, the temperature elevation facilitates the deformation of the AMFL and in situ drug release, substantially improving favorable delivery efficiencies of MSA-2 and its STING-stimulating activity on both cancer cell and DCs, thus triggering immune responses to switch the immunologically “cold” tumor to a more sensitive “hot” one. The synergistic photothermal-immunotherapeutic effect was verified through enhanced tumor regression, effective distant tumor suppression, and significant inhibition of postsurgical tumor recurrence and rechallenge. In summary, this thermoresponsive biomimicking nanomedicine operates precise PTT with real-time NIR-II imaging modality, which potentiates antitumor immune response by STING pathway activation for poorly immunogenic tumor treatment.

## Supporting Information Available

Experimental details and supporting results (PDF)
