# Supplementary material for: Pyridinium Rotor Strategy toward a Robust Photothermal Agent for STING Activation and Multimodal Image-Guided Immunotherapy for Triple-Negative Breast Cancer
Source: J Am Chem Soc. 2025 Feb 20;147(9):7433–44. doi: 10.1021/jacs.4c15534 (PMC11887044; doi:10.1021/jacs.4c15534)
Supplement: Supplementary file 1 — ja4c15534_si_001.pdf [file ja4c15534_si_001.pdf]

Supplementary Information

Pyridinium rotor strategy toward robust photothermal agent for STING activation and multimodal image-guided immunotherapy for triple-negative breast cancer

Shipeng Ning[*,†,‡], Ping Shangguan[†,§], Xinyan Zhu[†,||], Xinwen Ou[†,||], Kaiyuan Wang[¶,#,*], Meng Suo[§], Hanchen Shen[||], Xiuxin Lu[‡], Xianqing Wei[‡], Tianfu Zhang[*,§], Xiaoyuan Chen[*,#,††, ‡‡,§§], Ben Zhong Tang[*,||,||||]

[†]These authors contributed equally to this work.

*Corresponding authors. Email: nspdoctor@sr.gxmu.edu.cn; wangkaiyuan@hotmail.com (K.W.); zhangtf@gzhmu.edu.cn (T.Z.); chen.shawn@nus.edu.sg (X.C.); tangbenz@cuhk.edu.cn (B.Z.T.).

Materials and Experimental detail

Materials

Cholesterol and 1,2-dipalmitoyl-sn-glycero-3-phosphocholine (DPPC) were purchased from A.V.T (Shanghai, China). MSA-2 was purchased from Macklin (Shanghai, China). RPMI-1640 culture media was purchased from Wuhan Servicebio Technology Co., Ltd (China). Penicillin/streptomycin solution was purchased from New Cell & Molecular Biotech Co.,Ltd (Soochow, China). Fetal Bovine Serum was purchased from Nanjing BioChannel Biotechnology Co., Ltd (Nanjing, China). The ATP assay kit and HMGB1 ELISA kit (MDA) Content Assay Kit were purchased from Beijing Solarbio Science & Technology Co.,Ltd. (China). All of the aqueous solutions were prepared using purified deionized (DI) water purified with a purification system (Direct-Q3, Millipore, USA). The other solvents used in this work were purchased from Sinopharm Chemical Reagent (China) and Aladdin-Reagent (China).

Synthesis of the intermediates and the AIE molecules

TBTP was prepared according to the procedures described in the corresponding literature with modification (Scheme S1). The starting materials compound 1, compound 2 were purchased from Zhengzhou Ruke Biotechnology Co., Ltd.

To synthesis compound 3, $Pd(PPh_3)_4$ (58 mg, 0.05 mmol) and K_2CO_3 (483 mg, 3.50 mmol) were added to a solution of compound 1 (888 mg, 1.20 mmol) and compound 2 (431 mg, 1.00 mmol) in THF/water (v/v: 5:1) mixture (18 mL). Then the mixture was stirred at 70°C under N_2 for 4 h. And then the reaction was quenched by the addition of water and extracted with CH_2Cl_2 for 3 times. Solvent was removed by rotary evaporation. The product was purified by column chromatography (silica gel, CH_2Cl_2/hexane = 1:1, v/v) to obtain compound 3 as a light green solid.

To synthesis compound TBTP, $Pd(PPh_3)_4$ (30 mg, 0.05 mmol) and K_2CO_3 (242 mg, 3.50 mmol) were added to a solution of compound 3 (450 mg, 1.00 mmol) and 4-Pyridineboronic acid pinacol ester (115 mg, 1.20 mmol) in THF/water (v/v: 5:1) mixture (18 mL). Then the mixture was stirred at 70°C under N_2 for 12 h. And then the reaction was quenched by the addition of water and extracted with CH_2Cl_2 for 3 times. Solvent was removed by rotary evaporation. The product was purified by column chromatography (silica gel, CH_2Cl_2/MeOH = 20:1, v/v) to obtain compound TBTP as a dark green solid.

To synthesis compound TBTP-Bz, benzyl bromide (26 mg, 150 mmol) was added to a solution of TBTP (50 mg, 50 mmol) in THF/ACN (v/v: 1:3) mixture (4 mL). Then the mixture was stirred at 60°C under N_2 for 12 h. Solvent was removed by rotary evaporation. The raw product was redissolved in DMSO and precipitated by ethyl acetate for 3 times. The final product TBTP-Bz was dark brown solid. ^{1}H NMR (400 MHz, d_6-DMSO), δ (ppm): 9.12 (4H), 7.58 (4H), 7.48 (7H), 7.13 (4H), 6.98 (4H), 5.81 (2H), 3.77 (6H, –OCH3), 2.67 (2H), 2.33 (2H), 1.30 1.15 (m, 14H), 0.98-0.85 (16H). HRMS, m/z: calcd. for $C_{62}H_{65}BrN_6O_2S_4$: 1132.3235; found: 1132.3241.

To synthesis compound TBTP-COOH, similar procedures as that of TBTP-Bz was used by replacing benzyl bromide with 4-bromobenzoic acid (35 mg, 150 mmol). The final product TBTP-COOH was dark brown solid. ^{1}H NMR (400 MHz, d_6-DMSO), δ (ppm): 9.13 (2H), 8.97 (2H), 8.28 (1H), 8.19 (4H), 8.03 (3H), 7.64 (1H), 7.38 (1H), 7.09 (3H) 6.91 (5H), 5.91 (2H), 3.76 (6H, –OCH3), 2.95 (2H), 2.72 (2H), 1.29 1.16 (m, 14H), 0.87-0.78 (16H), HRMS, m/z: calcd. for $C_{63}H_{65}N_6O_4S_4^+$: 1097.3945; found: 1097.3953.

Characterization of AIE molecules

The UV-vis-NIR absorbance spectra were performed using Perkin Elmer Lambda 950 spectrophotometry (PerkinElmer, Inc.). The concentration of molecule were 1×10^{-5} M. The emission spectra of all three molecules was recorded under 808-nm diode laser excitation with concentration of 1 mM (HORIBA Co., Ltd). The Zeta potential of AIEgens was measured using Zeta potential and nano particle size instrument (Zetasizer Nano ZS90, Malvern Co., Ltd).

Photothermal Conversion Efficiency

An 808 nm NIR laser (Changchun New Industries Tech.Co., Ltd., Changchun, China) with irradiation powers was used to stimulate the different concentrations of AIE Aggregates solution. The photothermal images of suspensions under laser irradiation were captured using an infrared thermal imaging system (Fotric 225). The near-infrared (NIR) laser source was paired with a 4 mm diameter laser module featuring adjustable power settings. The photothermal conversion efficiency was determined using the following equation[2]:

$$\eta = \frac{hS(T_{max} - T_{surr}) - Q_0}{I(1 - 10^{-A_\lambda})}$$

where h is the heat transfer coefficient, S is the surface of the container, T_{max} and T_{surr} are the equilibrium temperature and ambient temperature, respectively. Q_0 is the heat associated with the light absorbance of the solvent, A_{λ} is the absorbance of AIE Aggregates solution at 808 nm, and I is the laser power density. According to the above equation, the η value of TBTP-Bz, for an representative, was determined to be about 58.5%.

MD simulations

To obtain amorphous aggregates in water, 40 AIEgen molecules were randomly placed in a cubic box with a side length of 10 nm. For the system containing positively charged molecules, requisite numbers of bromide ions were added to the box to neutralize the system. The energy minimization using the steepest descent algorithm was performed to relax the system. The aggregate configuration was then obtained from a 50 ns NVT ensemble simulation (T = 298.15 K). The water was then added to the simulation box to simulate the solvation environment of the aggregate. An equilibration procedure including a steepest descent minimization and a 50 ns NPT ensemble simulation (P = 1 atm and T = 298.15 K) was then performed to equilibrate the solvated system. After the equilibration of the solvated system, a 100 ns production run in NPT ensemble (P = 1 atm and T = 298.15 K) was performed to collect data.

The potential parameters of AIEgen molecules were taken from the General AMBER Force Field (GAFF), combined with the restrained electrostatic potential (RESP) charges, and the potential parameters of bromide ions were taken from the AMBER ff14SB Force Field, and the TIP3P model was chosen for water molecules. The V-rescale thermostat and the Berendsen barostat were chosen to control the system temperature and pressure, respectively. A typical cutoff distance of 1.2 nm was applied to calculate the short-range electrostatic interactions as well as the van der Waals interactions. The particle mesh Ewald (PME) method was employed to compute the long-range electrostatic interactions. The LINCS algorithm was employed to constrain the bonds involving hydrogen atoms. The periodic boundary conditions (PBC) were applied in all three dimensions of the simulation box. All the MD simulations were performed using the GROMACS 2020 package.

Quantum chemical calculations

For single molecule in solution, the ground-state (S_0) geometry was optimized using the DFT method at the M06-2X/6-31G(d) level. Grimme's DFT-D3 correction was utilized to describe the London dispersion effects. The solvent effect was considered using the polarizable continuum model (PCM) with water as solvent. The excited-state (S_1) geometry was optimized using the time-dependent density functional theory (TD-DFT) method at the same level of theory. We also calculated the ground-state (S_0) and excited-state (S_1) geometries of single molecule in gas phase to evaluate the reorganization energy, without considering solvent effect. For the aggregate state, the geometries were taken from the corresponding MD simulation structures, and then optimized based on an ONIOM model with the combined quantum mechanics (QM) and molecular mechanics (MM) methods. The innermost molecule was treated as the QM part and optimized at the (TD) M06-2X-D3/6-31G(d) level. The surrounding molecules were frozen to act as the MM part with the universal force field (UFF). The frequency calculations were carried out at the same level of theory to confirm that the optimized structure was at a minimum point of the potential energy surface. All the quantum chemical calculations were carried out using the Gaussian 16 software package. Reorganization energy analysis was carried out using the MOMAP package. The electrostatic potential analysis was conducted using the Multiwfn 3.8 software package.

Cell line

The 4T1 mouse breast cancer cell line was sourced from the Cell Bank of the Chinese Academy of Sciences and cultured in RPMI-1640 medium supplemented with 10% FBS in a humidified atmosphere at 37°C.

Animal tumor models

Female Balb/c aged 5-6 weeks were purchased from Vital River Company (Beijing, China). For the unilateral tumor model, Balb/c mice were subcutaneously injected with 5×10^6 4T1 cells into the right flank. For bilateral tumor models, Balb/c mice were subcutaneously injected with 5×10^6 4T1 cells into the right flank (primary tumors) and 1×10^6 4T1 cells into the left flank (distant tumors), respectively. The animal experiments were conducted in accordance with the protocol approved by the Ministry of Health in People's Republic of PR China and were approved by the Administrative Committee on Animal Research of Guangxi Medical University.

Comparison of AFL nanoparticles loaded with different AIE molecules

To compare the killing effect of AFL loaded with different AIE molecules on tumor cells, we prepared fusion liposome nanoparticles loaded with TBTP and TBTP-COOH using the same method as AFL loaded with TBTP-Bz, named AFLa and AFLc, respectively.

Then, 4T1 cells were incubated in six-well plates at 37 °C with 5% CO2 for 24 h; afterward, the culture medium was replaced by new culture medium, cells were incubated with 4 different groups: (1) PBS+NIR; (2) AFLa+NIR; (3) AFLb+NIR; (4) AFLc+NIR. AIE concentration was 0.2 mg/mL. Then, cells were exposed to 808 nm laser radiation (0.5 W/cm^2) for 5 min. After incubation for another 6 h. The viability of 4T1 cells was determined by a CCK-8 cell cytotoxicity assay according to the instructions.

The tumor-bearing mice were first divided randomly into 4 groups as above. 12 hours after injection, mice tumors were exposed to 808 nm laser radiation (0.5 W/cm^2) for 5 min. The AIE dose was 10 mg/kg. The temperature rise of the tumor site is measured by an infrared thermal image equipment.

Preparation and characterization of exosome Liposome hybrid nanoparticle loaded with TBTP-Bz and MSA-2 (AMFL)

The tumor exsomes derived from 4T1 cells were prepared according to the previous work. Subsequently, 27 mg DPPC, 2.8 mg Chol, 3 mg TBTP-Bz and 1mg MSA-2 were dissolved in chloroform, and then evaporated at 40°C for 80 min to form a thin film using a rotary evaporator. This film was then hydrated with a PBS solution, subjected to ultrasound, and extruded multiple times through 100 nm polycarbonate pores with the addition of EXO (3 mg protein). The resulting AMFL particles were dialyzed overnight in a dialysis bag (MWCO 300 kDa). The loading capacities of TBTP-Bz and MSA-2 were calculated using HPLC. Loading capacity = M_{drug}/M_{AMFL}, where M refers to the mass. Liposomal DPPC loaded with TBTP-Bz and MSA-2 (AML) were prepared similarly, with the exception of excluding exsomes. The loading efficiency of MSA-2 is 11.3±1.6% and that of TBTP-Bz is 72.7±2.6%. The hybrid exosome-liposomes loaded with TBTP-Bz (AFL) were

prepared similarly, excluding MSA-2. Blank hybrid exosome-liposomes (FL) were also prepared similarly, excluding both TBTP-Bz and MSA-2. The morphology of the nanoparticles was examined using TEM (JEOL-2100). Protein expression was assessed through western blot analysis. Particle size and zeta potential were determined using DLS, and key proteins were identified using western blotting. The protein content on EXO in AMFL was quantified using the BCA Protein Assay Kit (Beyotime Biotech. Inc.). DSC was performed using a differential scanning calorimeter (DSC-Q2000).

Drug release study

Briefly, HPLC was performed by using Agilent HPLC1260 II under the test wavelength of 280 nm. The sample was dissolved in methanol, applied on an Agilent C18 column (3.5 μm, 100 × 4.6 mm), and eluted at 1 mL/min from acetonitrile (0.1% TFA, gradient from 10% to 100%). Flow rate: 1.0 mL/min, Column Temperature: 25°C, Injection Volume: 10 μL.

In vitro cancer targeting study

4T1 cells were seeded in 24-well plates and cultured for 12 h. Then, 100 μL DiO labeled AML or AMFL (containing 30 μg mL^{-1} TBTP-Bz) was added to the medium. Then, the cells were incubated for 1h at 37 °C and 5% CO_2 and washed with PBS three times. The cells were harvested, stained with DAPI (Beyotime Biotech. Inc. Shanghai, China) imaged by using a CLSM (Zeiss LSM 880).

Induction of ICD with AMFL

After seeded in 48-well plate (2 × 10^4 cells per well) for 12 h, 4T1 cells were pre-incubated incubated and treated with 6 different groups: (1) PBS+NIR (0.5 W cm^{-2}, 5 min); (2) FL; (3) AMFL; (4) AML+NIR; (5) AFL+NIR and (6) AMFL+NIR. The concentration of TBTP-Bz was 0.2 mg mL^{-1}. The PTT was conducted 12 h after nanoparticles treatment. The cells were then washed with PBS three times, fixed with 4% PFA and permeabilized with 0.1% Triton X-100 for 10 min. After washed with PBS three times, the cells were blocked with 10% FBS, and incubated with Anti-Calreticulin/FITC Conjugated antibody (Bioss) for 30 min. The cells were washed with PBS three times, then stained with DAPI for 20 min. Finally, the cells were washed with PBS three times and observed using CLSM.

4T1 cells were seeded into the 12-well plate (2 × 10^5 cells/well) and the next day the cells were treated with 6 different groups: (1) PBS+NIR (0.5 W cm^{-2}, 5 min);

(2) FL; (3) AMFL; (4) AML+NIR; (5) AFL+NIR and (6) AMFL+NIR. The concentration of TBTP-Bz was 0.2 mg mL^{-1}. The PTT was conducted 2 h after nanoparticles treatment. After 24 h, the expression of HMGB1 was detected through CLSM. For quantification of released HMGB1 in medium, the medium was collected after the cells treated with materials. Then 20 μL medium was used for ELISA detection of various cytokines and ATP levels according to the kit instructions (Elabscience Biotechnology Co.,Ltd.).

Transwell experiment on DCs stimulation in vitro

BMDCs were isolated from 8-week-old Balb/c mice bone marrow. For BMDCs maturation assay, 1×10^5 4T1 cells were treated by the above groups and then cocultured with 1×10^6 BMDCs in the transwell culture system. Then BMDCs were stained with anti-CD80, and anti-CD86 (Biolegend). Finally, the cells were sorted using flow cytometer (Beckman-Coulter, USA). The cytokines secreted by BMDCs were detected by ELISA according to the kit instructions (Elabscience Biotechnology Co.,Ltd.).

In vitro anti-cancer effect of AMFL

Typically, 4T1 cells were incubated in six-well plates at 37 °C with 5% CO_2 for 24 h; afterward, the culture medium was replaced by new culture medium, cells were incubated with (1) PBS+NIR (0.5 W cm^{-2}, 5 min); (2) FL; (3) AMFL; (4) AML+NIR; (5) AFL+NIR and (6) AMFL+NIR. The concentration of TBTP-Bz was 0.2 mg mL^{-1}. The PTT was conducted 2 h after nanoparticles treatment. After incubation for another 48 h, the viability of 4T1 cells was determined by a CCK-8 (Beyotime Biotech. Inc.) according to the instructions. The toxicity of AMFL at different AIE concentrations was also tested using a similar method.

Cellular apoptosis was assessed by plating 4T1 cells in 6-well plates and treating them via the five methods listed above for 12 h. After an additional 12 h, cells were harvested with EDTA-free trypsin, and annexin V-FITC/PI (Elabscience Biotechnology Co.,Ltd. Wuhan, China) was used to analyze cellular apoptosis via flow cytometry.

Western blot (WB) analysis in vitro

4T1 cells (8×10^6 per plate) were incubated with (1) PBS+NIR (0.5 W cm^{-2}, 5 min); (2) FL; (3) AMFL; (4) AML+NIR; (5) AFL+NIR and (6) AMFL+NIR. The concentration of TBTP-Bz was 0.2 mg mL^{-1}. The PTT was conducted 2 h after

nanoparticles treatment. After 12 hours of incubation, the cells of each group were collected, the protein lysis solution was added to extract the total cell protein, and the protein was quantified according to the instructions of the BCA reagent. After electrophoresis, the separated proteins were transferred to PVDF membranes. After the film was transferred, the milk was blocked for 2 hours, and the diluted primary Anti-Phospho-TBK1 antibody (Bioss), Anti-phospho-IRF3 antibody (Bioss) or anti-Phospho-STING rabbit antibody (Cell Signaling Technology) solution (diluted according to the recommended concentration of each antibody instruction) was added overnight. After washing, the secondary antibody was added, exposed, developed, and filmed. BMDCs were processed and detected using similar methods as described above.

In vivo NIR-II FLI

To monitor the in vivo photothermal effect, AML and AMFL (100 μL, containing 10 mg kg^{-1} TBTP-Bz) was intravenously injected into the tumor-bearing mice, respectively, and then subjected to in vivo imaging system (Artemis Intelligent Imaging, Shanghai, China) using 808 nm excitation and 1250 nm long-pass filter.

In vivo IR thermography

To monitor the in vivo photothermal effect, AMFL (100 μL, containing 10 mg kg^{-1} TBTP-Bz) was intravenously injected into the tumor-bearing mice, and then the tumors suffered from 0.5 W cm^{-2} irradiation for 5 min at 12 h post-injection. PBS injection used as control group.

In Vivo PA imaging

The 4T1 tumor-bearing mice were intravenously injected with AMFL (100 μL, containing 10 mg kg^{-1} TBTP-Bz). At different time points after injection, photoacoustic imaging was performed on a PA imaging system (VEVO LAZR).

In vivo anti-bilateral tumor study

Female Balb/c aged 5-6 week were purchased from Vital River Company (Beijing, China). Balb/c mice were subcutaneous injected with 5×10^6 4T1 cells into the right flank (primary tumors) and 1×10^6 4T1 cells into the left flank (distant tumors), respectively. When the tumor grew to approximately 200 mm^3, treatment was carried out on day 0. The mice were firstly divided randomly into 6 different groups (Each group included 5 mice): (1) PBS+NIR (0.5 W cm^{-2}, 5 min);

(2) FL; (3) AMFL; (4) AML+NIR; (5) AFL+NIR and (6) AMFL+NIR. The PTT was conducted 12 h after nanoparticles treatment. The dose of TBTP-Bz was 10 mg kg^{-1}. Mice body weight and tumor volume in all groups were monitored every 3 days. A caliper was employed to measure the tumor length and tumor width, and the tumor volume was calculated according to following formula. Tumor volume = tumor length × tumor $width^2$ / 2. After 21 days treatment, mice were sacrificed. Five main organs (heart, liver, spleen, lung and kidney) of all mice were harvested, washed with PBS, and fixed with paraformaldehyde for histology analysis. Collect mouse blood for biochemical analysis. And the tumor tissues were weighed, and fixed in 4% neutral buffered formalin, processed routinely into paraffin, and sectioned at 4 μm. The primary tumor sections were stained with HE, Anti-Phospho-TBK1 FITC Conjugated antibody (Bioss) or Anti-phospho-IRF3 FITC Conjugated antibody (Bioss) and finally examined by using fluorescence microscope (IX81, Olympus, Japan). The distant tumor sections were stained with anti-CD8 and HE and finally examined by using fluorescence microscope (IX81, Olympus, Japan) after the end of treatment.

To examine DC maturation in vivo, the spleens were harvested. The frequency of DC maturation was then examined by CD11c+ cell sorting kit (ThermoFisher) and flow cytometry after immunofluorescence staining with FITC-anti-CD80 and PE-anti-CD86 (Abcam, China). For flow cytometric analysis in lymph nodes, collected lymph nodes (LNs) were ground using a 200-mesh filter to prepare corresponding single cells suspensions. The frequency of DC maturation in the LNs was then examined by $CD11c^+$ cell sorting kit (Thermo Fisher Scientific, USA) and flow cytometry after immunofluorescence staining with FITC-anti-CD80 and PE-anti-CD86 (Abcam). The frequency of $CD8^+$ T cells in LNs was then examined by $CD45^+$ cell sorting kit (Thermo Fisher Scientific, USA) and flow cytometry after immunofluorescence staining with FITC-anti-CD3 and PE-anti-CD8 (Abcam). To study the T cells content and function in tumors, tumors were harvested from mice in different groups and then examined by $CD3^+$ cell sorting kit (ThermoFisher) treated with flow cytometry after immunofluorescence staining with FITC-anti-CD4 and PE-anti-CD8 (Abcam, China); To analysis treatment-induced cytokine secretion, primary tumors were collected from mice. The concentration of proinflammatory cytokines including TNF-α, IFN-β and IFN-

γ were then analyzed with ELISA kits (Elabscience Biotechnology Co.,Ltd. China) according to the manufacturer's instructions.

Assessment of inflammatory factors in mouse blood

Female Balb/c aged 5-6 week were purchased from Vital River Company (Beijing, China). Balb/c mice were subcutaneous injected with 3 × 10^6 4T1 cells into the right flank. When the tumor grew to approximately 200 mm^3, treatment was carried out on day 0. The mice were firstly divided randomly into 3 different groups (Each group included 3 mice): (1) PBS; (2) MSA-2; (3) AMFL. The dose of MSA-2 was 1.6 mg kg^{-1}. The free MSA-2 was orally administrated. PBS and AMFL were intravenously injected. Blood samples were collected from mice on days 1, 3, and 7 after injection, the levels of TNF-α and IFN-β in the peripheral blood samples were measured according to the manufacturer's instructions.

Biodistribution

To study the biodistribution of AML and AMFL in various organs, Balb/c mice bearing 4T1 tumor xenografts (n = 3) received an intravenous (i.v.) injection of 100 μL PBS containing AML or AMFL (with an equivalent TBTP-Bz dose of 10 mg/kg). Tumor tissues and main organs (heart, liver, spleen, lung and kidney) were collected at 12 h after the injection and all these tissues were performed by ultrasonic tissue destructor and suspended in acetonitrile before subjecting to vigorous homogenation and filtration. Then, the TBTP-Bz concentration were measured by high performance liquid chromatography (HPLC). Briefly, HPLC was performed by using Agilent HPLC1260 II under the test wavelength of 254 nm. The sample was dissolved in acetonitrile, applied on a Kromasil C18 column (10 μm, 250 x 4.6 mm) from Teknokroma, and eluted at 2 mL/min with a 70 min gradient from acetonitrile (0.1% TFA, gradient from 5% to 95%)/ methanol mixture.

Assessment of inflammatory factors in mouse blood

Female Balb/c aged 5-6 weeks were purchased from Vital River Company (Beijing, China). Balb/c mice were subcutaneously injected with 3 × 10^6 4T1 cells into the right flank. When the tumor grew to approximately 200 mm^3, treatment was carried out on day 0. The mice were firstly divided randomly into 3 different groups (Each group included 3 mice): (1) PBS; (2) MSA-2; (3) AMFL. The dose of MSA-2 was 1.6 mg kg^{-1}. The free MSA-2 was orally administrated. PBS and AMFL

were intravenously injected. Blood samples were collected from mice on days 1, 3, and 7 after injection, the levels of TNF-α and IFN-β in the peripheral blood samples were measured according to the manufacturer's instructions.

Quantitative RT-qPCR.

Total RNA was isolated using the RNA-Quick Purification Kit (Esscience) and cDNA was reverse transceibed using the PrimeScript™ RT reagent Kit (TaKaRa). Quantitative RT-qPCR was performed using TB Green Premix Ex Taq II (Takapa) on an QuantStudio™ 5 Real-Time PCR system (ThermoFisher Scientific, USA). For all gene expression data beta-Actin was used as an endogenous normalisation control. Primer sequences are as follows: Gapdh forward, 5'-GGCCTTCCGTGTTCCTACC-3', Gapdh reverse, 5'-AGCCCAAGATGCCCTTCAGT-3', Ifnb1 forward, 5'-TCCTGCTGTGCTTCTCCACCACA-3', Ifnb1 reverse, 5'-AAGTCCGCCCTGTAGGTGAGGTT-3', Il6 forward, 5'-CTGCAAGAGACTTCCATCCAG-3', Il6 reverse, 5'-AGTGGTATAGACAGGTCTGTTGG-3'.

In vivo antitumor recurrence and rechallenge study

Female BALB/c aged 5-6 week were purchased from Vital River Company (Beijing, China). Balb/c mice were subcutaneous injected with 5×10^6 4T1 cells into the right flank. The mice were firstly divided randomly into 6 different groups (Each group included 5 mice): (1) PBS+NIR (0.5 W cm^{-2}, 5 min); (2) FL; (3) AMFL; (4) AML+NIR; (5) AFL+NIR and (6) AMFL+NIR. The PTT was conducted 12 h after nanoparticles treatment. The dose of TBTP-Bz was 10 mg kg^{-1}. Then the primary tumors were resected mice at 4 days post first treatment, and the mice were rechallenged a secondary 4T1 tumor at the left hind leg. The growth of recurrence and rechallenge tumor was recorded at certain time intervals. A caliper was employed to measure the tumor length and tumor width, and the tumor volume was calculated according to following formula. Tumor volume = tumor length × tumor width^2 / 2. After 21 days treatment, mice were sacrificed. The blood samples from these mice were collected for blood biochemistry analysis and immune memory study. The live $CD3^+CD8^+$ T lymphocytes were isolated by using the Mouse $CD3^+$ and $CD8^+$ T Cell Isolation Kit (NovoBiotechnology Co., LTD. China), stained with antibodies (anti-CD62L and anti-CD44). The subpopulations of T cells were finally analyzed on a flow cytometer. The tumor tissues were weighed, and fixed in 4% neutral buffered formalin, processed routinely into paraffin, and

sectioned at 4 μm. Then the tumor sections were stained with H&E and TUNEL and finally examined by using fluorescence microscope (IX81, Olympus, Japan).

Statistical analysis

Data analyses were conducted using the GraphPad Prism 8 software. For variance analysis, One-way analysis of variance (ANOVA) with Tukey's post hoc test was used. p values of <0.05 were considered significant. $^*p < 0.05$, $^{**}p < 0.01$, $^{***}p < 0.001$, and $^{****}P < 0.0001$.

Supporting results

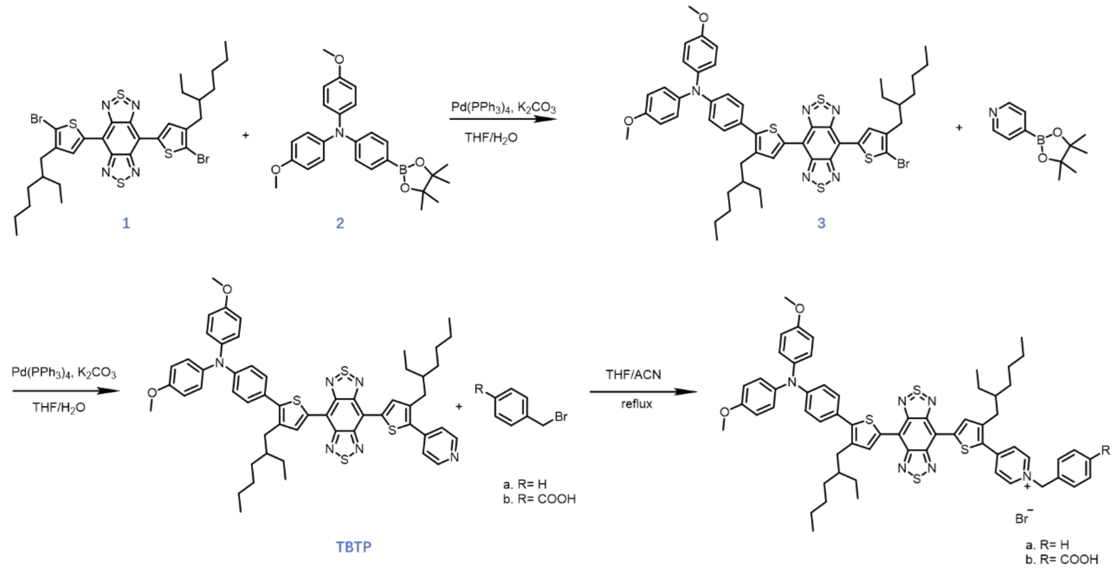

Scheme S1. Synthetic route to TBTP-Bz and TBTP-COOH.

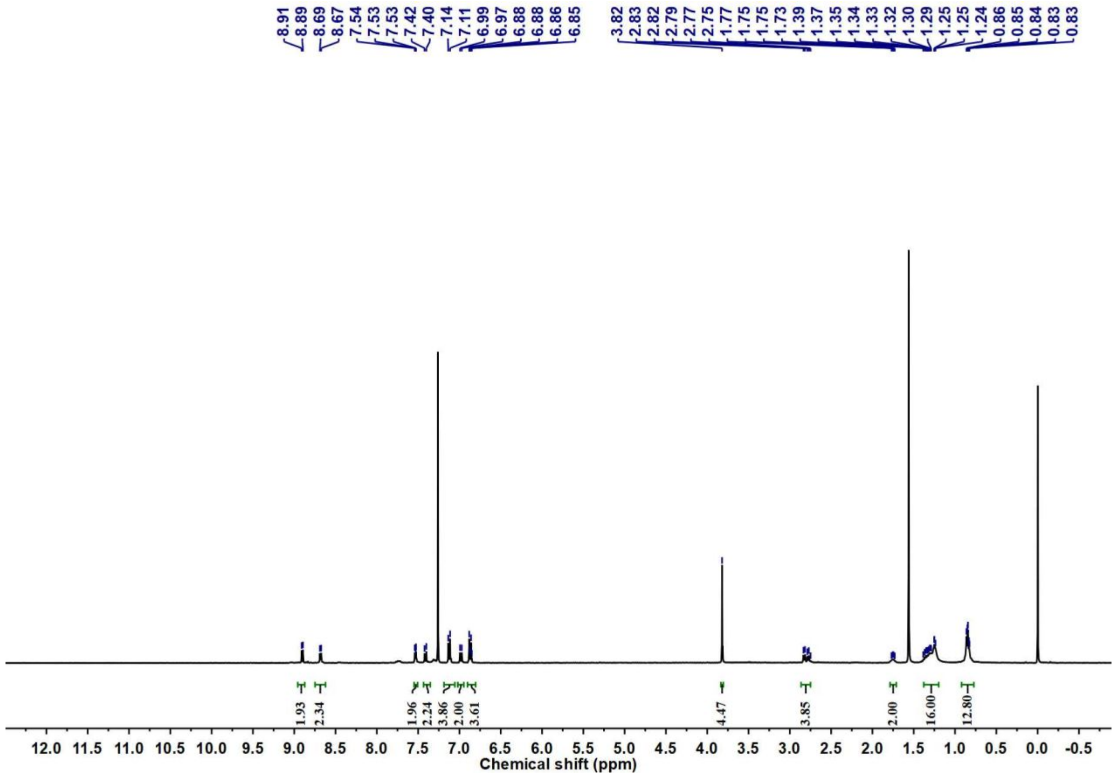

Figure S1. ^{1}H NMR spectrum of TBTP.

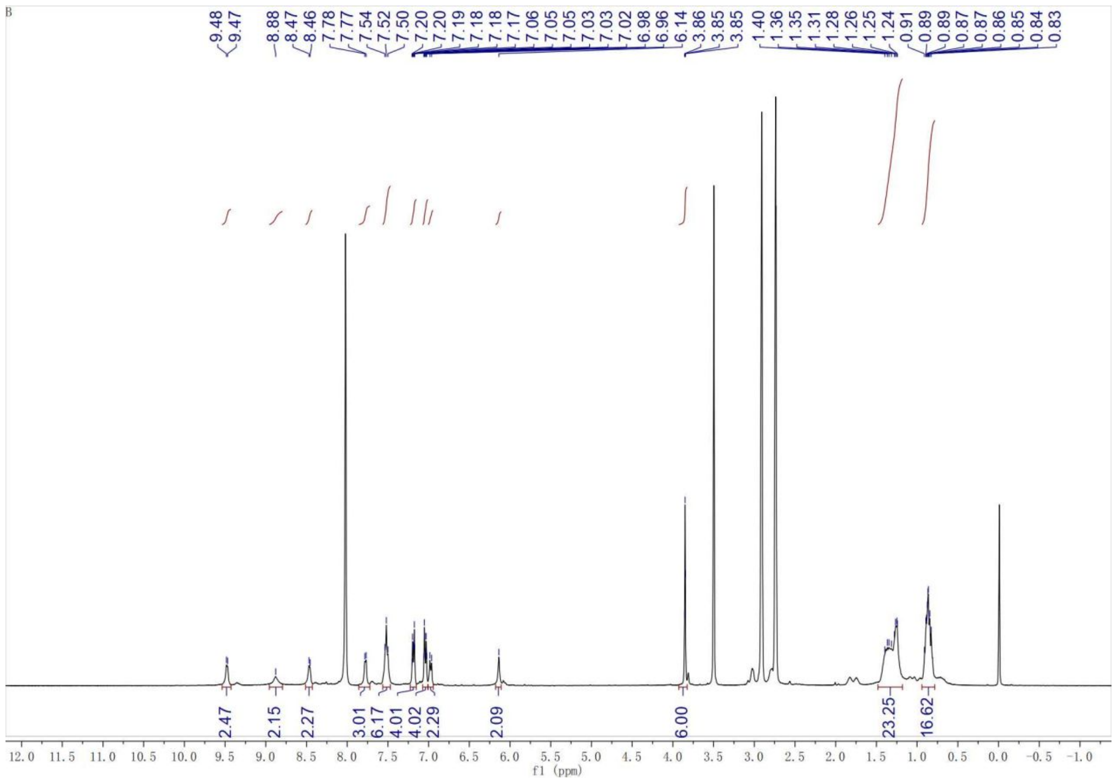

Figure S2. ^{1}H NMR spectrum of TBTP-Bz.

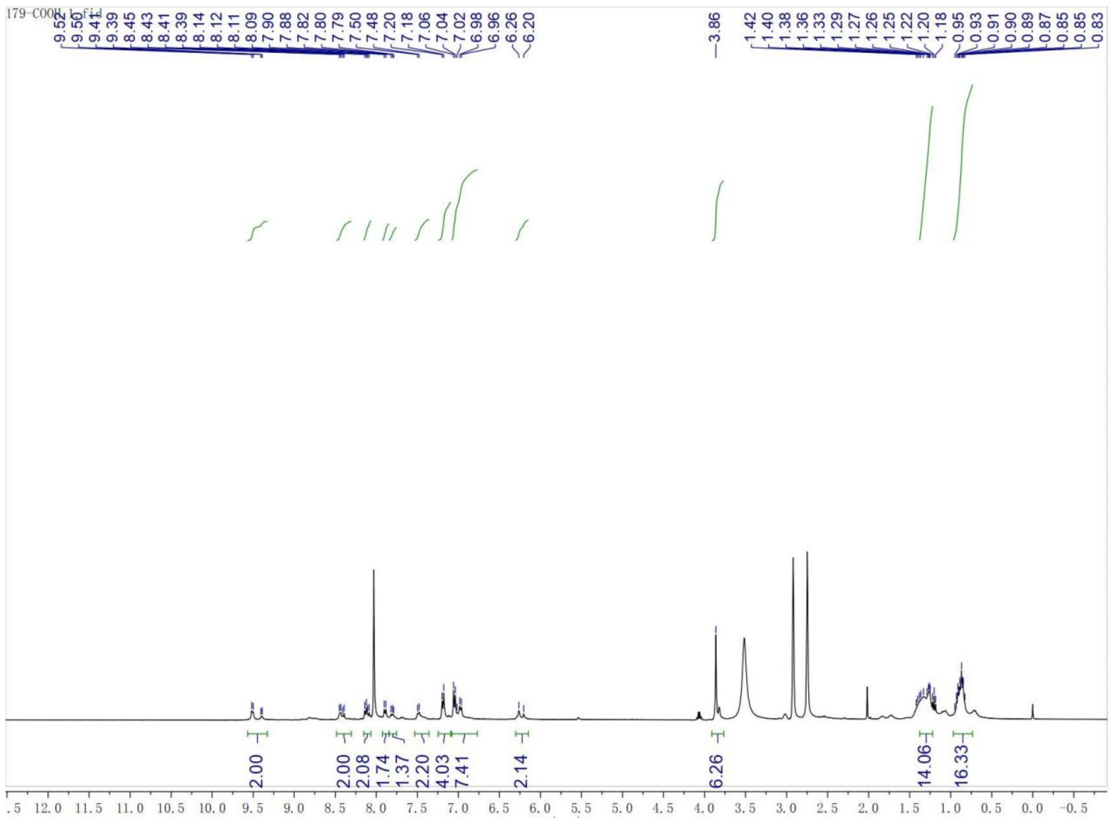

Figure S3. ^{1}H NMR spectrum of TBTP-COOH.

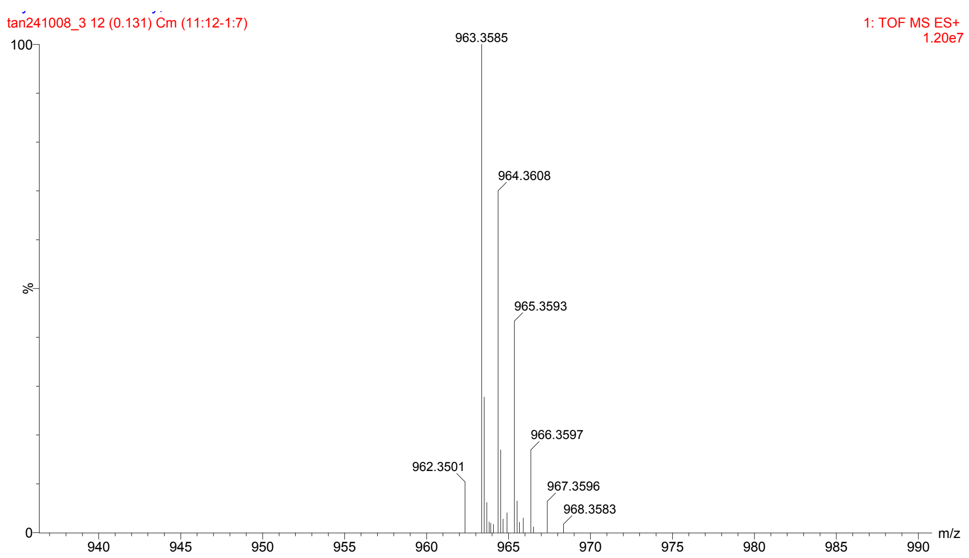

Figure S4. HRMS spectrum of TBTP.

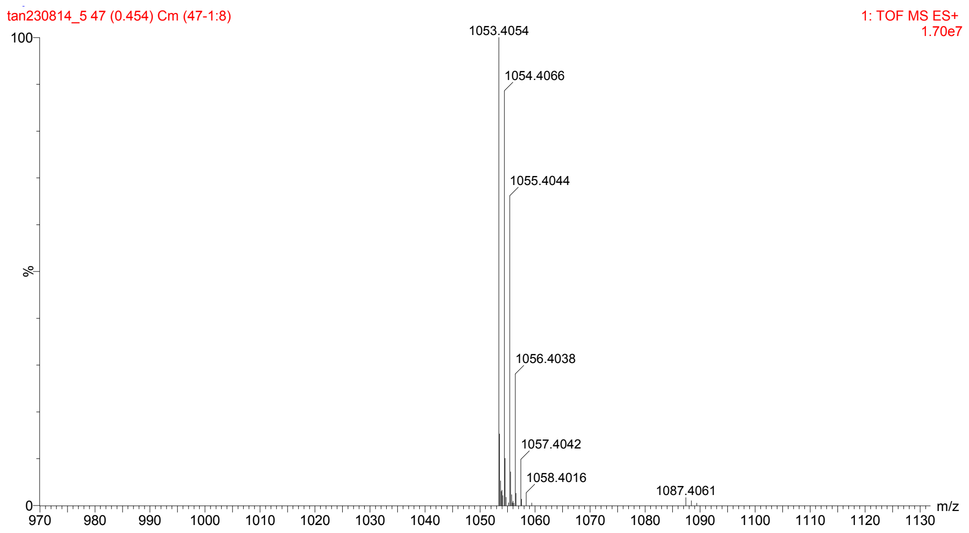

Figure S5. HRMS spectrum of TBTP-Bz.

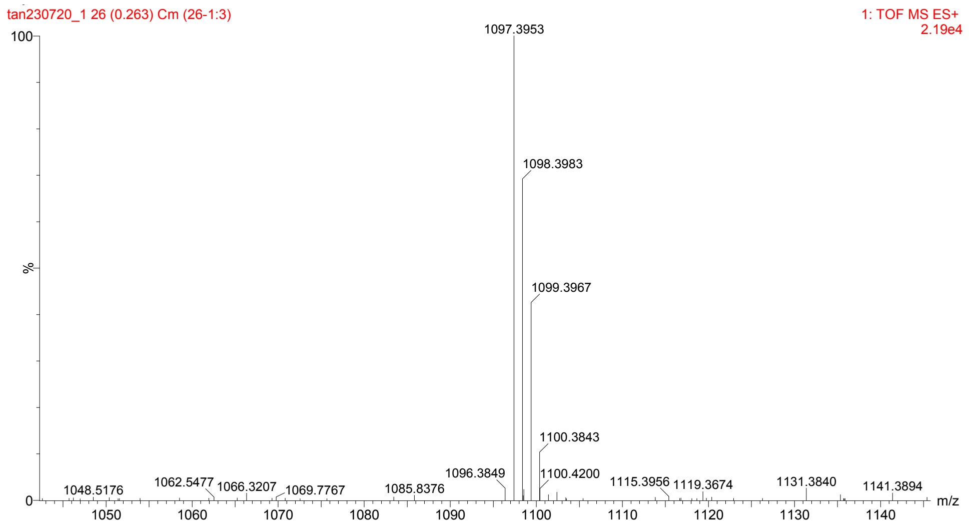

Figure S6. HRMS spectrum of TBTP-COOH.

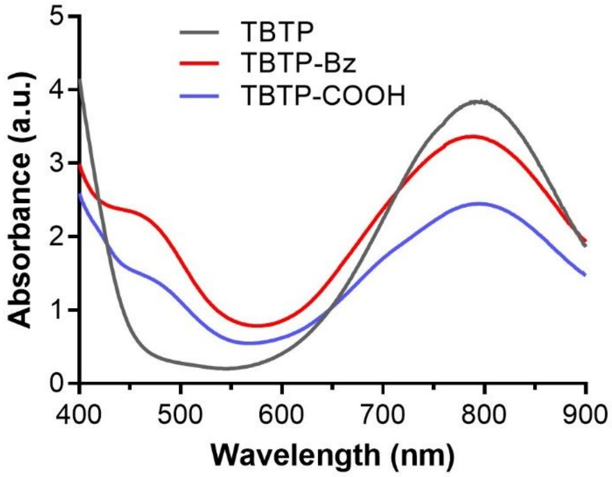

Figure S7. The absorbance of TBTP, TBTP-Bz and TBTP-COOH in water. The concentration of the molecules: 1.5 x 10^{-4} M.

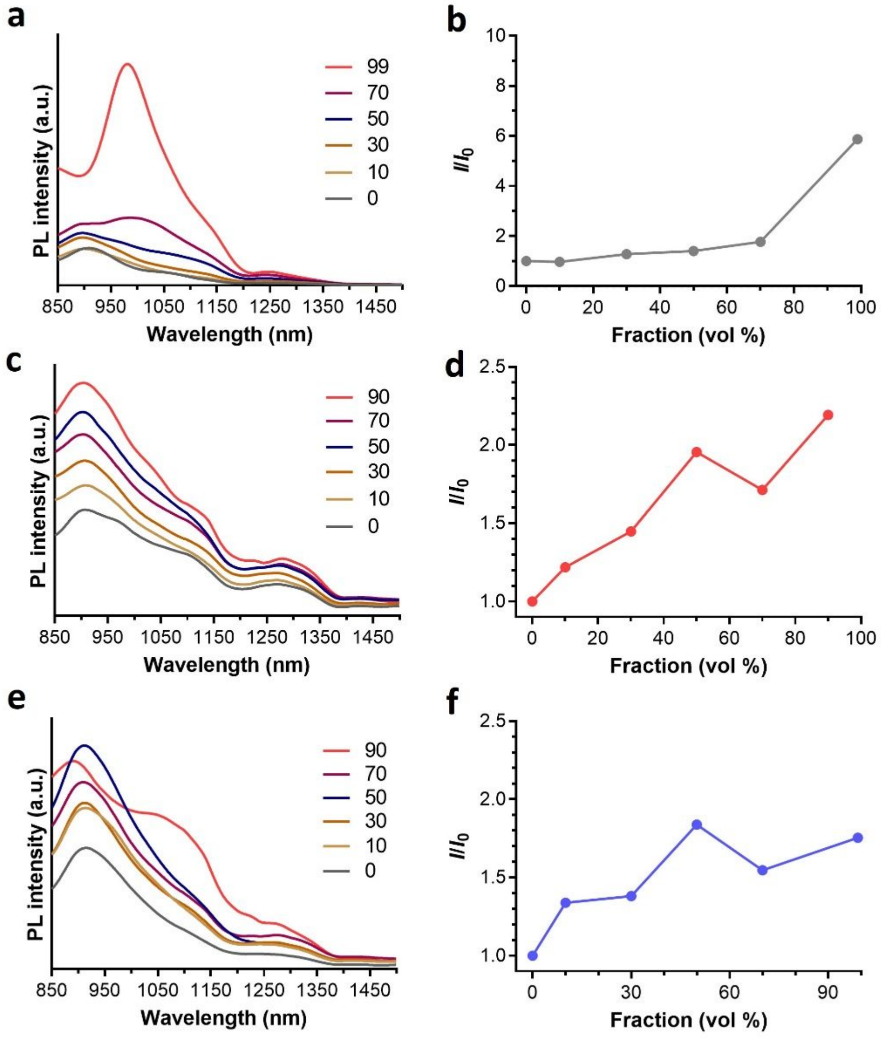

Figure S8. (a) Photoluminescence (PL) spectra of TBTP in THF/hexene mixtures at different hexene fractions (volume percentage, vol %). Molecular concentration: 10^{-5} M; λ_{ex} = 808 nm. (b) Plots of relative PL intensity of TBTP at its PL maximum (I/I_0) versus hexene fractions. I_0 = emission intensity in pure THF (0%). (c) PL spectra of TBTP-Bz in DMF/diethyl ether mixtures at different hexene fractions (vol %). Molecular concentration: 10^{-5} M; λ_{ex} = 808 nm. (d) Plots of relative PL intensity of TBTP-Bz at its PL maximum (I/I_0) versus diethyl ether fractions. I_0 = emission intensity in pure DMF (0%). (e) PL spectra of TBTP-COOH in DMF/diethyl ether mixtures at different hexene fractions (vol %). Molecular concentration: 10^{-5} M; λ_{ex} = 808 nm. (f) Plots of relative PL intensity of TBTP-COOH at its PL maximum (I/I_0) versus diethyl ether fractions. I_0 = emission intensity in pure DMF (0%).

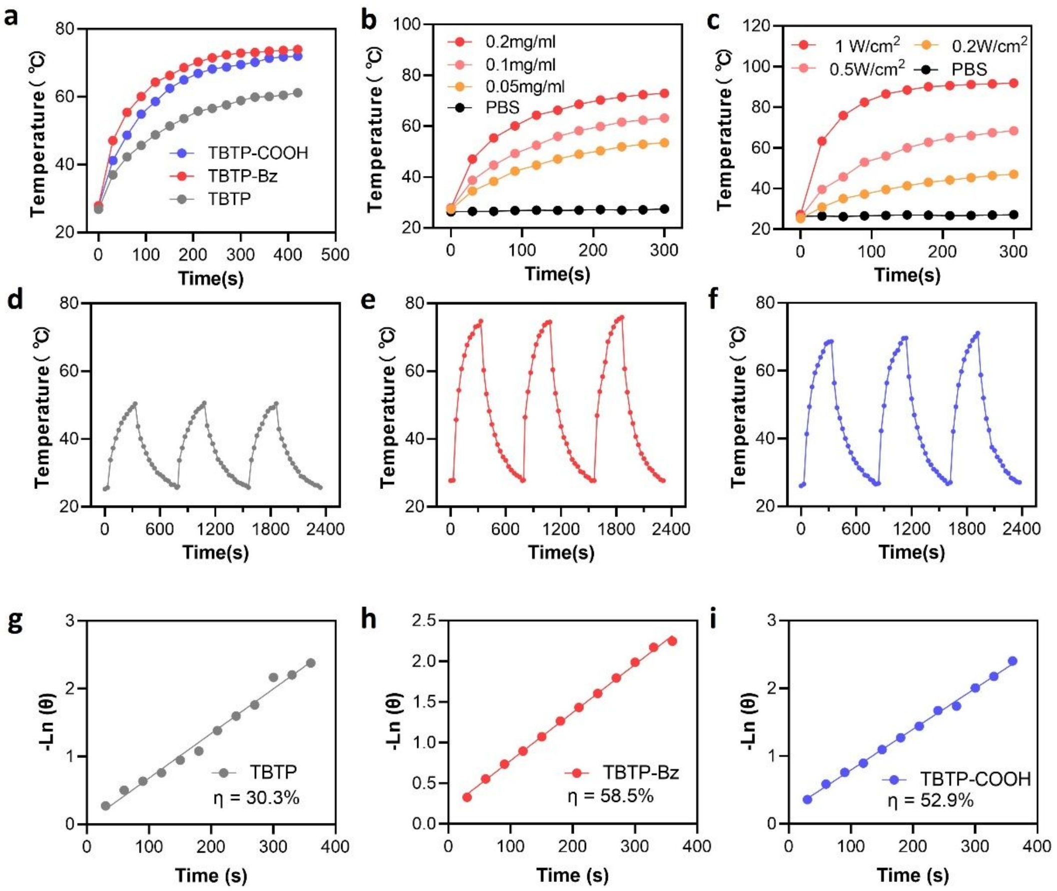

Figure S9. (a) The temperature curves with different AIE molecules in PBS solution (0.2 mg mL^{-1}) under 808 nm laser irradiation (0.5 W cm^{-2}). (b) The temperature curves of TBTP-Bz with different concentrations under 808 nm laser irradiation (0.5 W cm^{-2}). (c) The temperature curves of TBTP-Bz (0.2 mg mL^{-1}) under 808 nm laser irradiation with different power density. (d-f) Photothermal stability of TBTP, TBTP-Bz and TBTP-COOH aggregates (0.2 mg mL^{-1}) upon 808 nm laser irradiation of 0.5 W cm^{-2} for three on/off cycles. (g-i) Linear correlation of the negative natural logarithm of driving force temperatures versus cooling times for different AIE molecules.

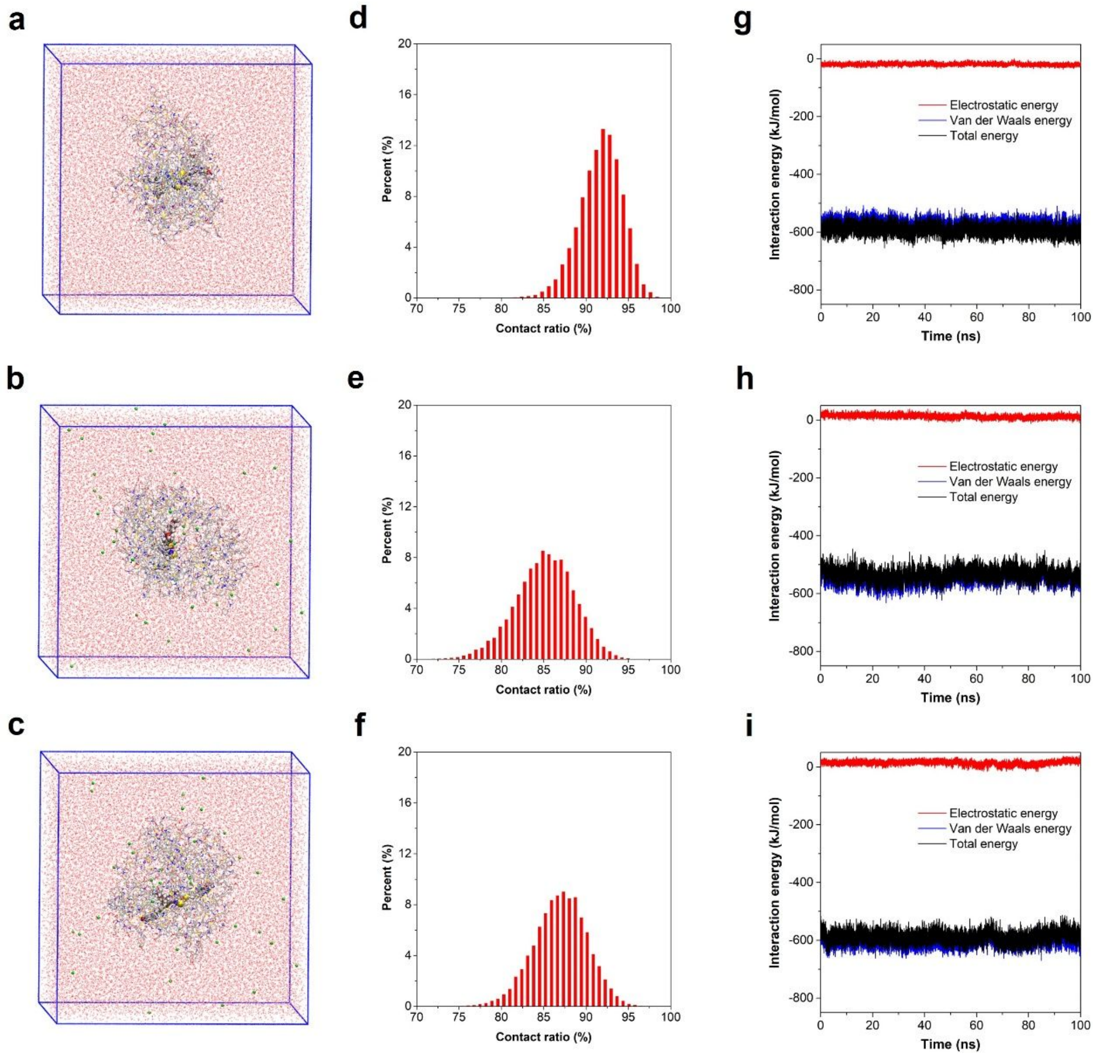

Figure S10. (a-c) The simulation model of TBTP, TBTP-Bz and TBTP-COOH aggregate in water. (d-f) Atomic contact ratio distribution of the innermost molecule with surrounding organic molecules in TBTP, TBTP-Bz and TBTP-COOH aggregate obtained from the whole simulation trajectory. (g-i) The interaction energy (both electrostatic energy and Van der Waals energy components) of the innermost molecule with surrounding organic molecules in TBTP, TBTP-Bz and TBTP-COOH aggregate.

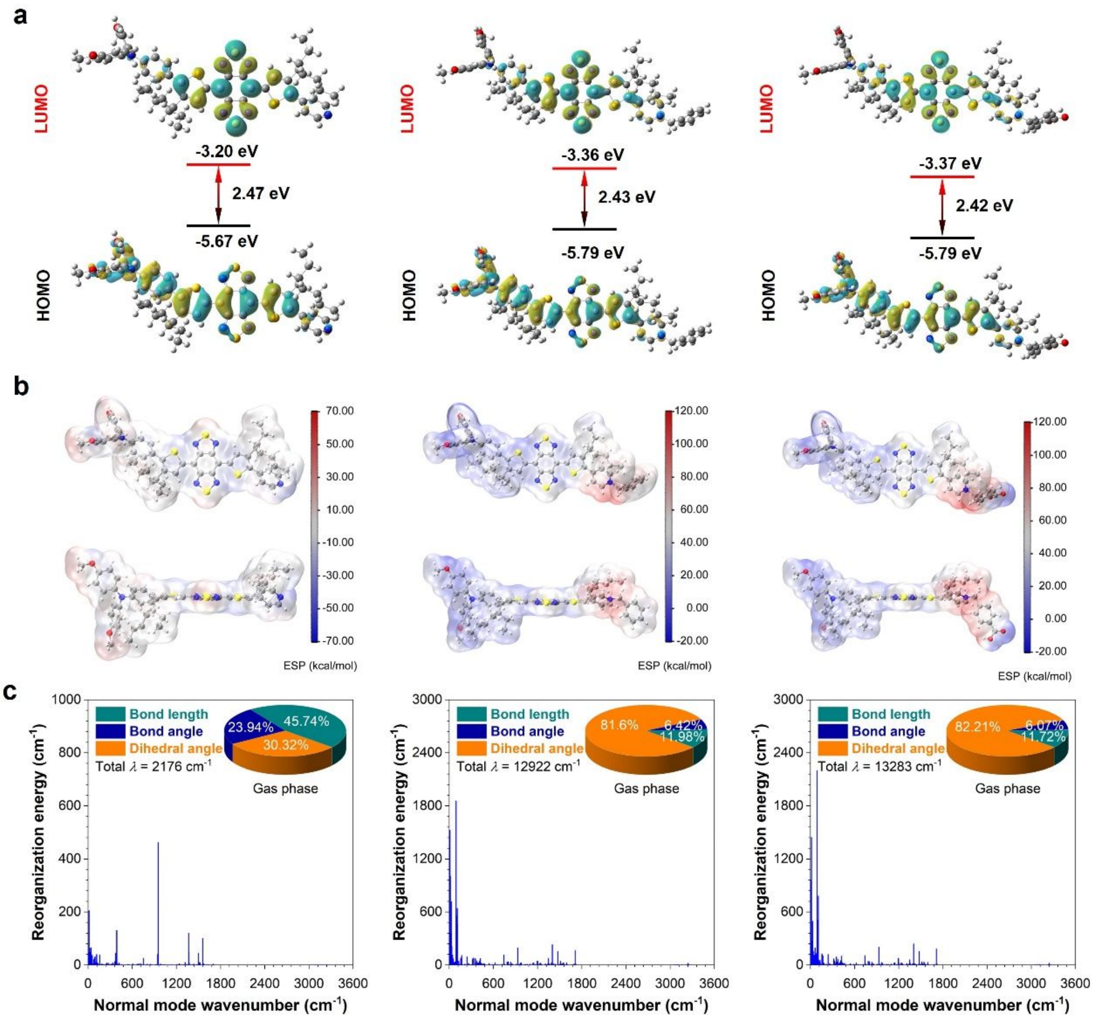

Figure S11. (a) Frontier molecular orbitals of TBTP, TBTP-Bz and TBTP-COOH based on the optimized excited-state (S_1) geometry in implicit water solvent. (b) The molecular electrostatic potential map of TBTP, TBTP-Bz and TBTP-COOH molecule based on the optimized excited-state (S1) geometry in implicit water solvent. (c) Plots of reorganization energy *vs* normal mode wavenumber of TBTP, TBTP-Bz and TBTP-COOH in gas phase. Inset: the proportions of bond length, bond angle, and dihedral angle contributed to the total reorganization energy.

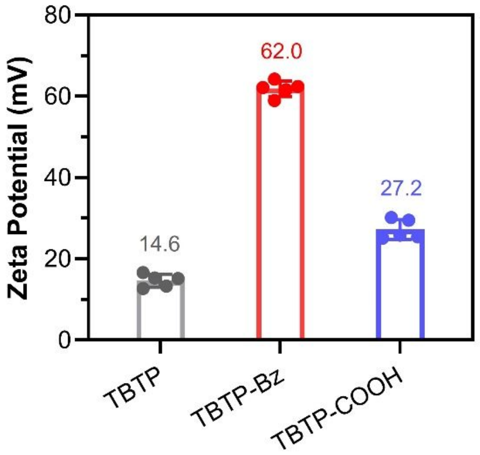

Figure S12. The Zeta potential of TBTP, TBTP-Bz and TBTP-COOH in deionized water. Molecular concentration: 10^{-5} M.

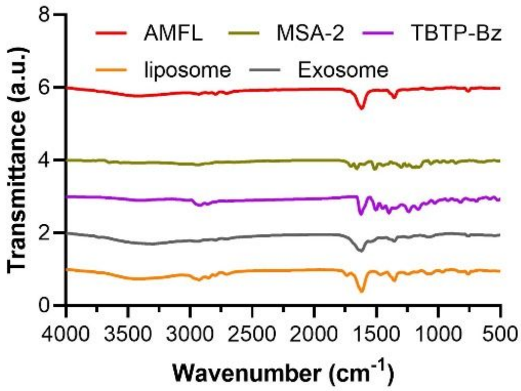

Figure S13. FTIR spectrum of AMFL, TBTP-Bz, MSA-2, Liposome and 4T1 cells derived exosome membrane.

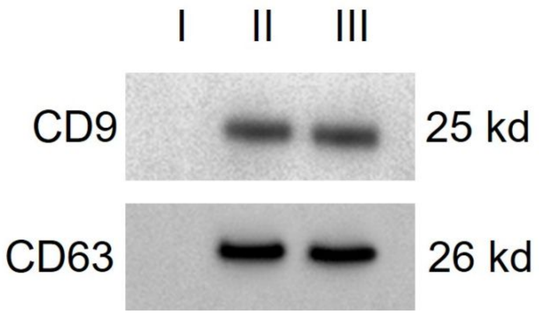

Figure S14. Markers of different nanoparticles or vesicles detected by WB. I: pure liposome. II: 4T1 cancer cells derived exosome. III: AMFL.

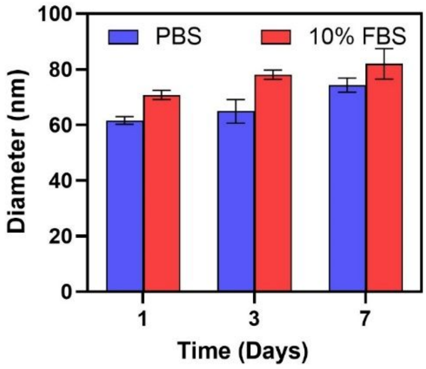

Figure S15. Diameters of AMFL incubated in PBS or 10% FBS for different times, measured by DLS. n = 3.

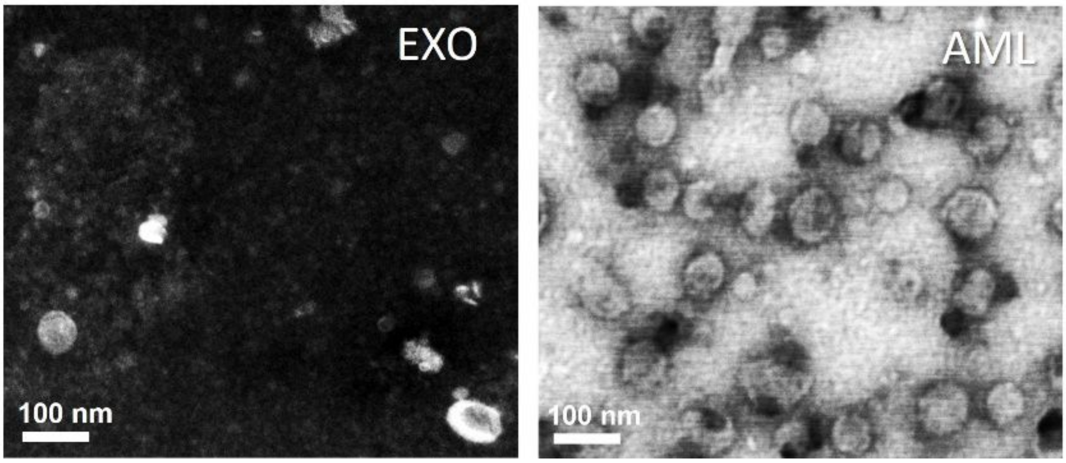

Figure S16. TEM images of various nanoparticles. Scale bars: 100 nm.

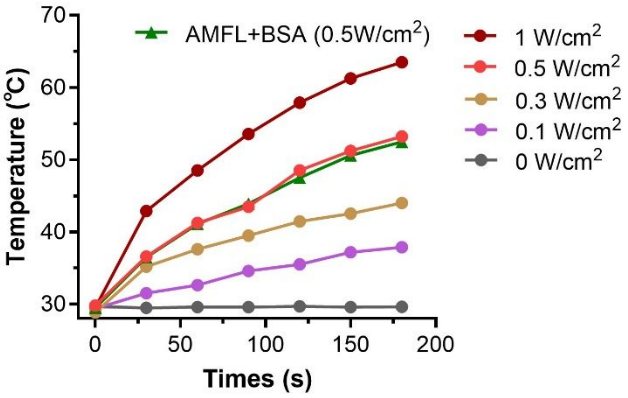

Figure S17. Temperature change curves of AMFL with different under NIR laser exposure with different power densities (808 nm, 100 μg/mL). To investigate the photothermal behavior of AMFL in the presence of proteins, we conducted the experiment in BSA solution (30 mg/mL BSA). n = 3.

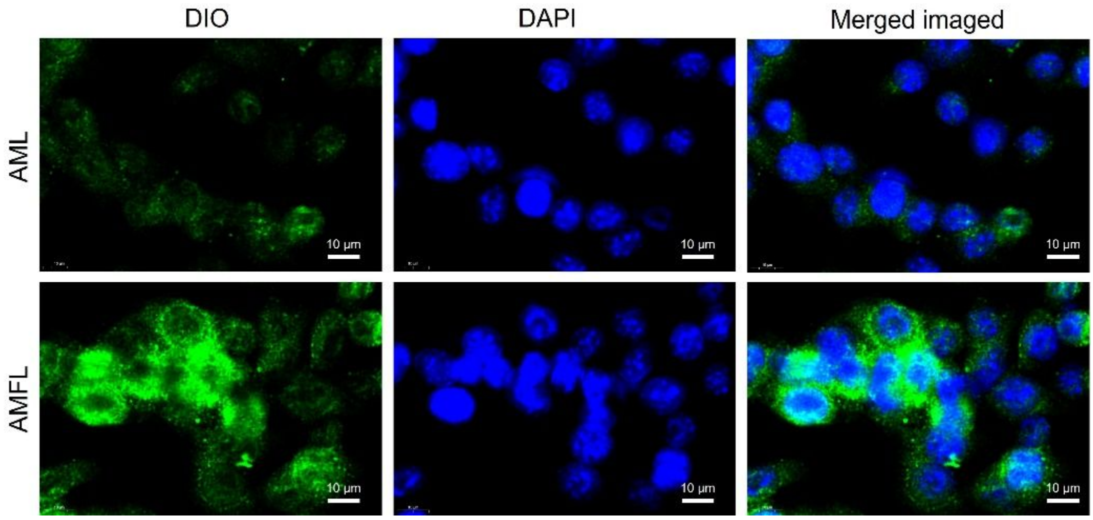

Figure S18. CLSM image of 4T1 cells incubated with AML and AMFL (containing 30 μg mL^{-1} TBTP-Bz) for 12 h. AML and AMFL were labeled with green fluorescent membrane dyes, 3,3′-dioctadecyloxacarbocyanine perchlorate (DIO). The cells were then harvested and co-stained with 4',6-diamidino-2-phenylindole (DAPI). Scale bars: 10 μm.

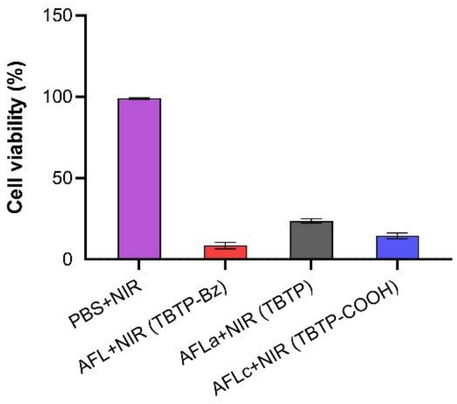

Figure S19. Cell cytotoxicity of AFLa, AFL and AFLc upon laser irradiation were determined kept, n = 3.

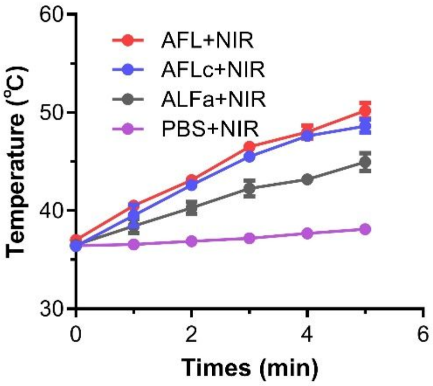

Figure S20. Corresponding quantification curve from *in vivo* photothermal effect at 12 h post-injection with AFL, AFLa or AFLc upon laser irradiation (808 nm, 0.5 W/cm^2) in 5 min. Data are presented as mean ± SD. *n* = 3.

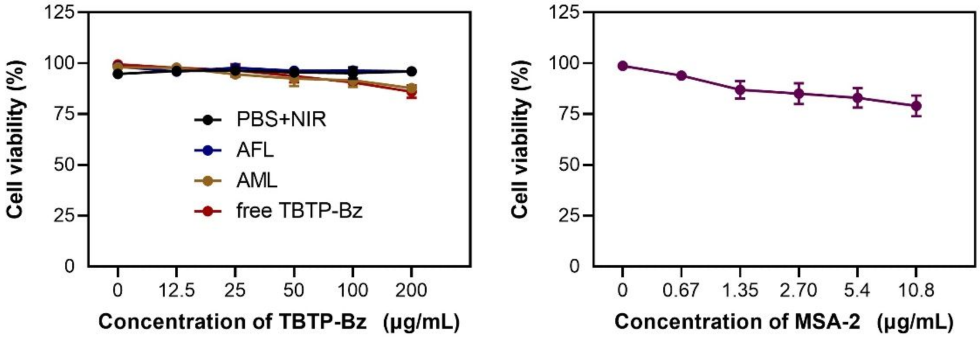

Figure S21. Cell cytotoxicity after co-incubating with AML, AFL, free TBTP-Bz and MSA-2. The concentrations of AML and AFL are based on the TBTP-Bz concentration, n = 3.

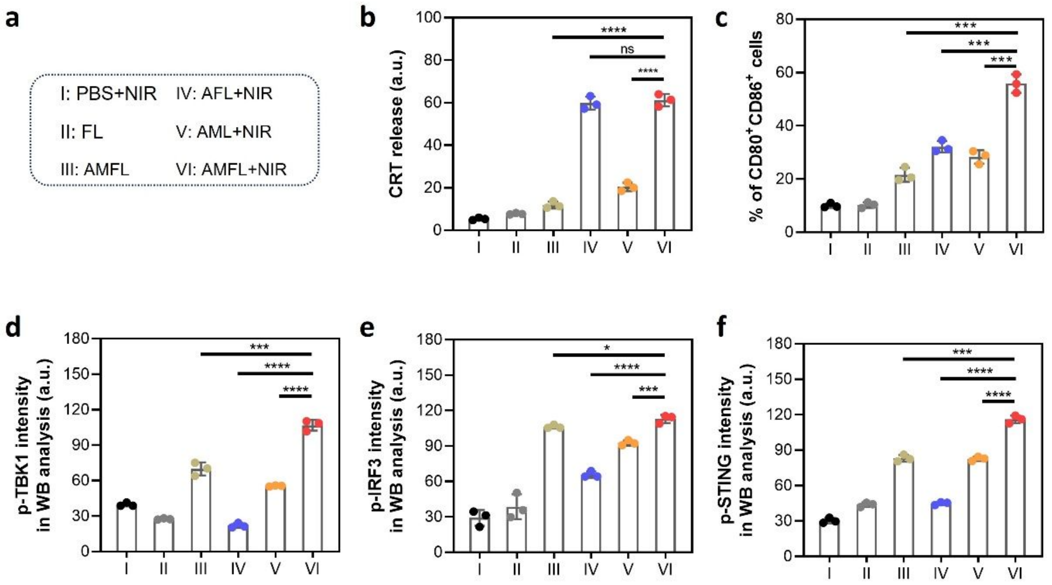

Figure S22. (a) The in vitro experimental groups. (b) The fluorescent intensity of CRT signal in 4T1 cells after different treatment as shown in Figure 4d. (c) Percentage of mature DC ($CD80^+CD86^+$) after incubating with 4T1 cells lysis under different treatment. (d-f) Relative WB gray intensity of p-TBK1, p-IRF3 and p-STING in Fig. 4f. Data are shown as mean ± SD with n = 3. The gray intensity was analyzed by software Image J. Statistical significance was determined using one-way analysis of variance (ANOVA): *P < 0.05, **P < 0.01, ***p < 0.001, ****p < 0.0001, ns denotes no significant difference.

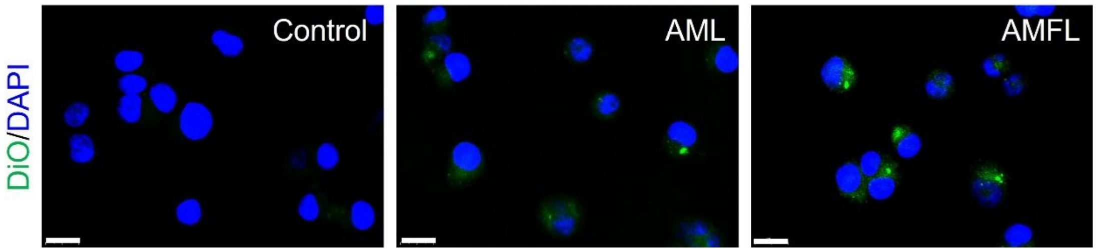

Figure S23. Incubation of DiO-labeled AML or AMFL with DC cells. Scale bars: 20 μm.

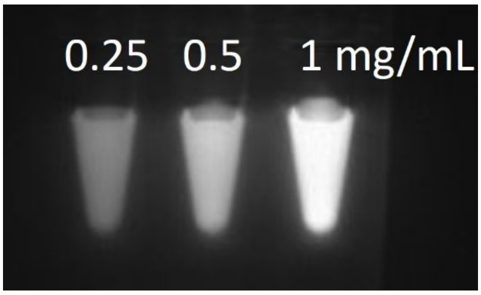

Figure S24. NIR-II imaging of various concentrations of TBTP-Bz in AMFL under 1000 nm long-pass filters. λ_{ex} = 808 nm.

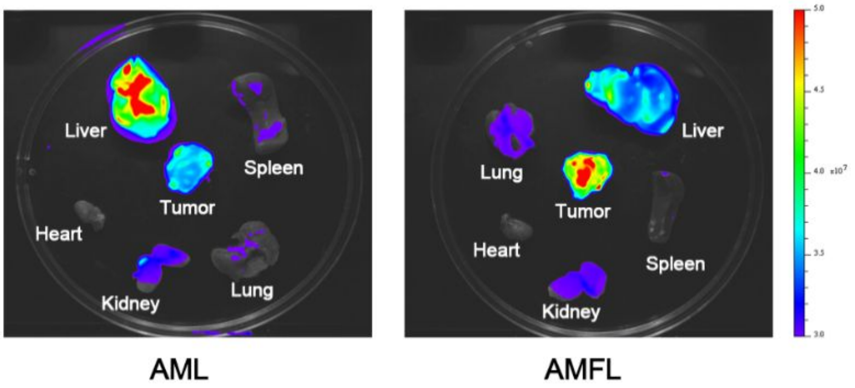

Figure S25. *Ex vivo* fluorescence images of 4T1 tumor-bearing mice after intravenous injection of 100 μL DiD-labeled AML or AMFL (with an equivalent TBTP-Bz dose of 10 mg/kg) after 12 h. λ_{ex} = 644 nm, λ_{em} = 665 nm. n = 3.

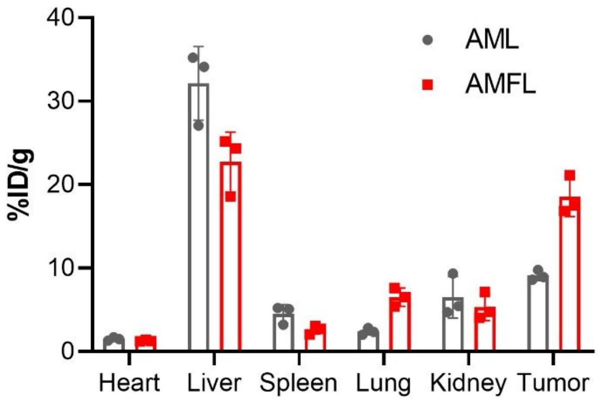

Figure S26. Biodistribution of main organs and tumor tissues of mice after receiving an intravenous injection of 100 μL PBS containing AML or AMFL for 12 h. n =3.

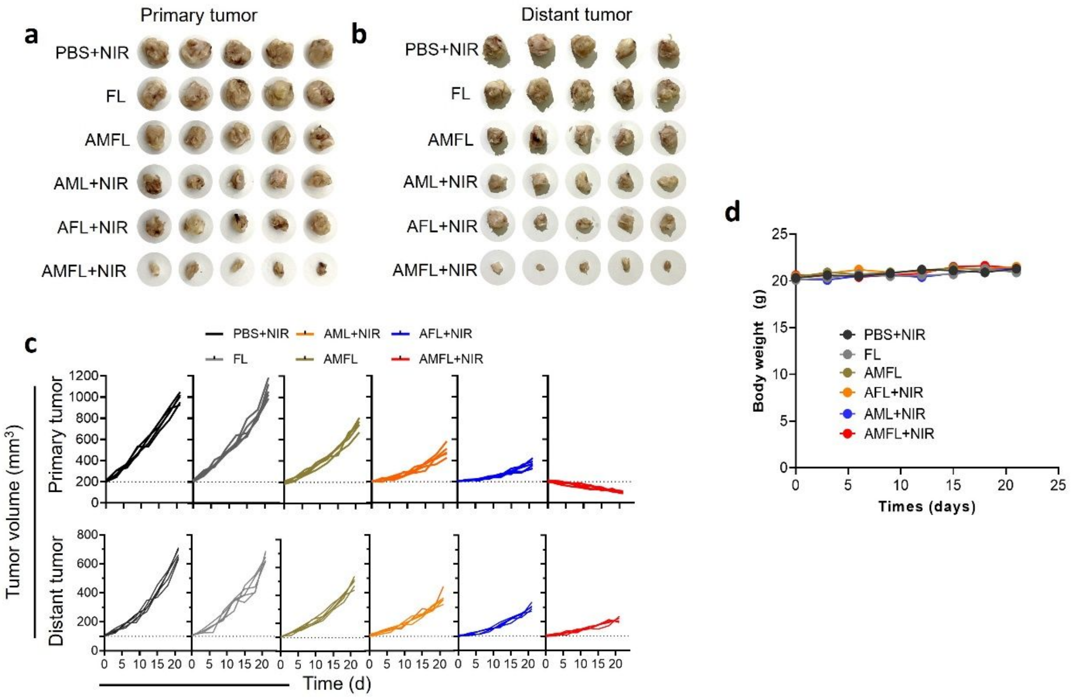

Figure S27. Supplement data of Figure 6 in the manuscript. (a) Photographs of primary tumors and (b) distant tumors collected from sacrificed mice after different treatments on day 21. (c) Growth curves of primary and distant 4T1 tumors treated as indicated. The horizontal dotted line indicates the initial tumor volume. (d) Body weight curves of mice with different treatments.

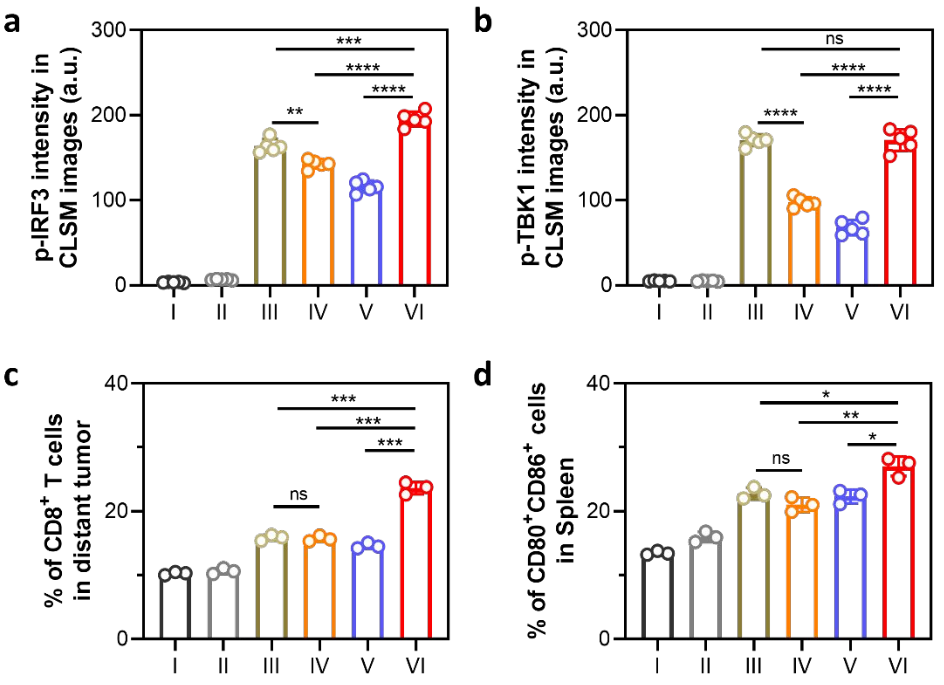

Figure S28. (a and b) The fluorescent intensity of p-IRF3 and p-TBK1 signal in the slices of primary tumor after different treatment as shown in Fig. 7a, n = 5. (c) Percentage of $CD8^+$ T cells in distant tumor measured in Fig. 7c, n = 3. (d) Percentage of mature DC ($CD80^+CD86^+$) in spleen measured in flow cytometry results in Fig. 7b, n = 3. Statistical significance was determined using one-way analysis of variance (ANOVA): *P < 0.05, **P < 0.01, ***p < 0.001, ****p < 0.0001, ns denotes no significant difference.

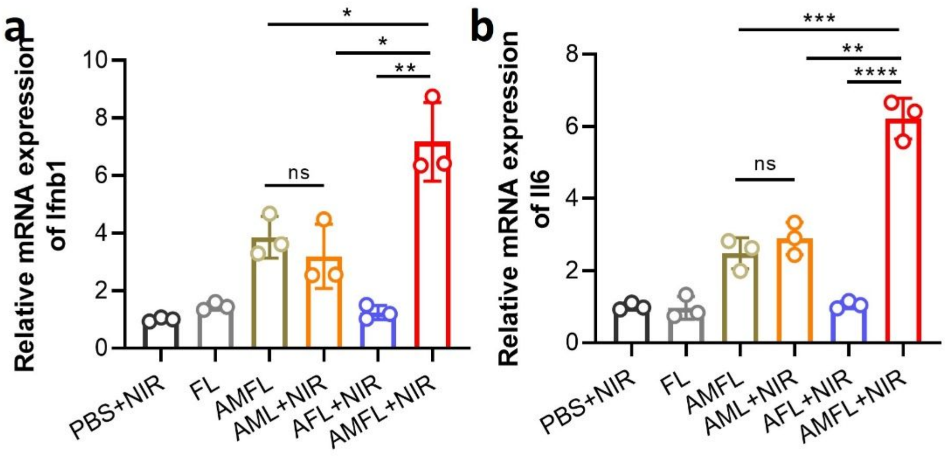

Figure S29. Quantification of abundance of mRNAs from tumor tissues for the indicated genes by RT-qPCR after the according in vivo anti-bilateral tumor treatment, $n = 3$. Statistical significance was determined using one-way analysis of variance (ANOVA): *P < 0.05, **P < 0.01, ***p < 0.001, ****p < 0.0001, ns denotes no significant difference.

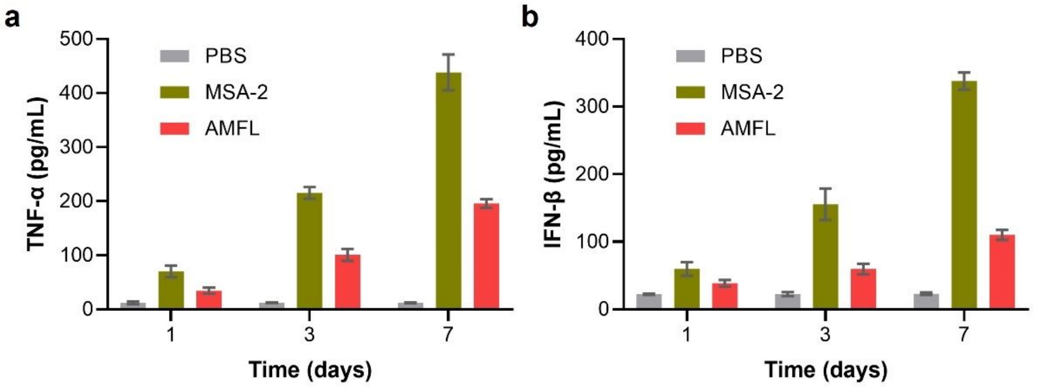

Figure S30. The TNF-α and IFN-β levels in the peripheral blood samples collected from tumor-bearing mice after being administered with different formulations. The free MSA-2 was orally administrated. PBS and AMFL were intravenously injected. n = 3.

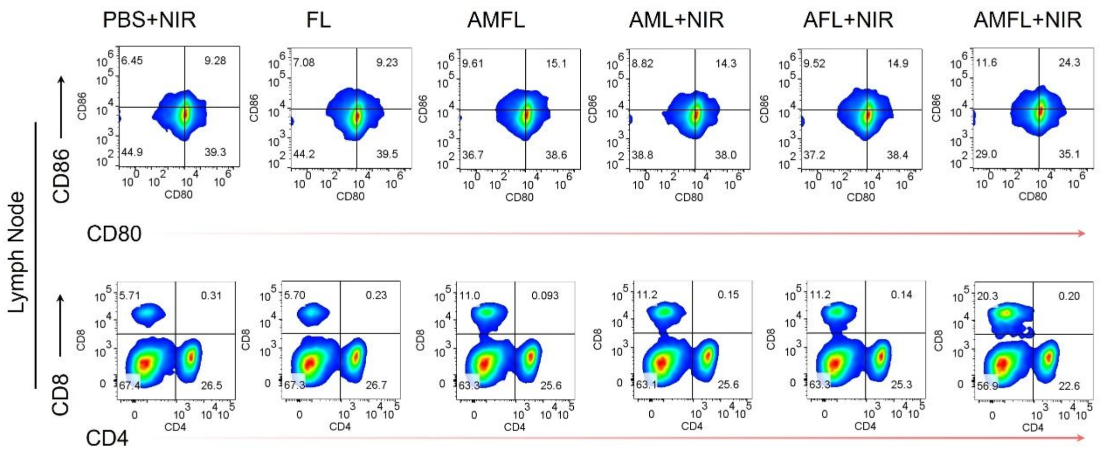

Figure S31. Upper row: Flow cytometry analysis of DC maturation (CD80/CD86) in lymph nodes after different treatments. Lower row: Flow cytometry analysis of T cell activation (CD4/CD8) in lymph nodes after different treatments. n = 3.

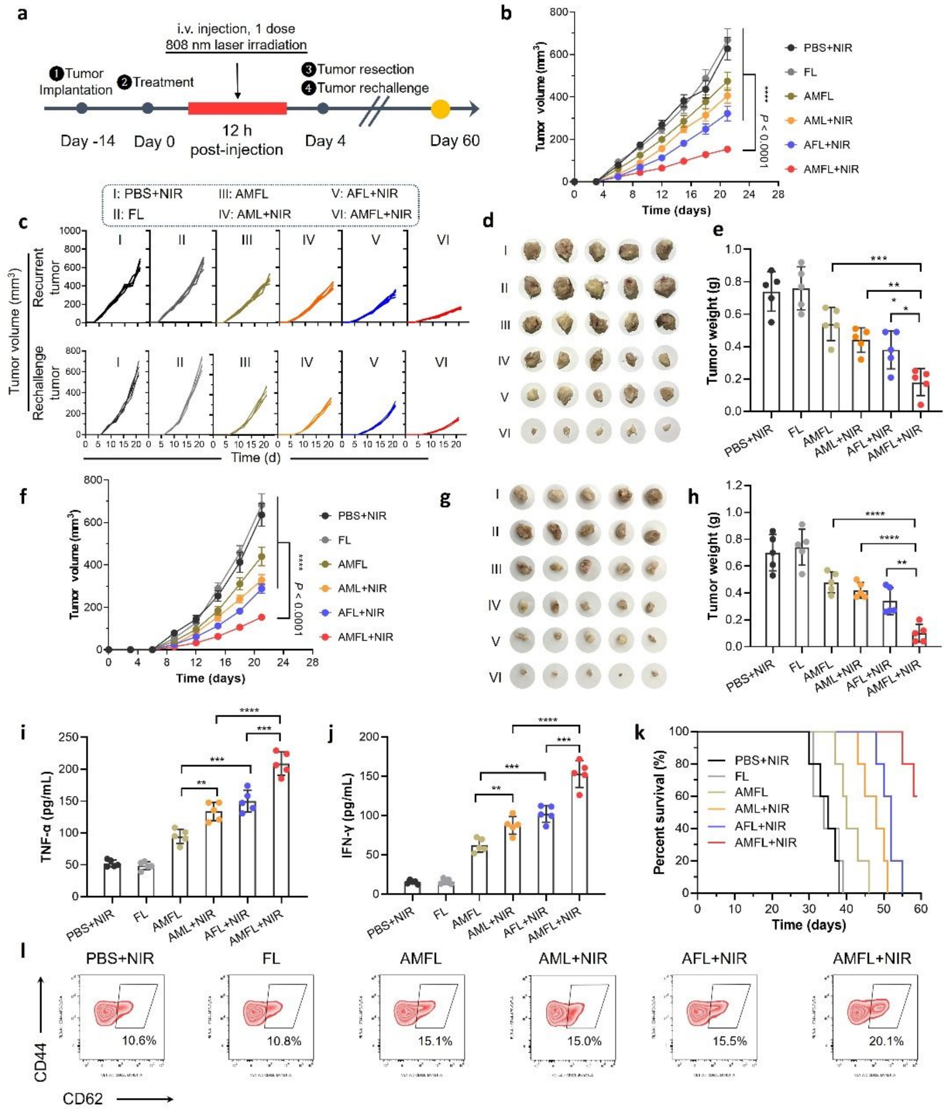

Figure S32. AMFL elicits systemic immunity to suppress tumor recurrence and rechallenge. (a) Schematic illustration of the treatment procedures. (b) Tumor volume of recurrent tumor over time after injection of different treatments. (c) Growth curves of recurrent and rechallenge 4T1 tumors treated as indicated. (d) Photograph and (e) tumor weight of recurrent tumors collected from sacrificed mice after different treatments on day 21. (f) Tumor volume of rechallenge tumor over time after injection of different treatments. (g) Photograph (h) and tumor weight of rechallenge tumors collected from sacrificed mice after different treatments on day 21. ELISA results of (i) TNF-α, and (j) IFN-γ in tumor tissue under different treatments. (k) Survival of mice after different treatment. (l) Flow cytometry analysis of TCM (CD44/CD62) activation. Data were given as mean ± S.D. (n = 5) Statistical significance was calculated via one-way ANOVA with Tukey's test: *p < 0.05; **p < 0.01; ***p < 0.001, ****p < 0.0001

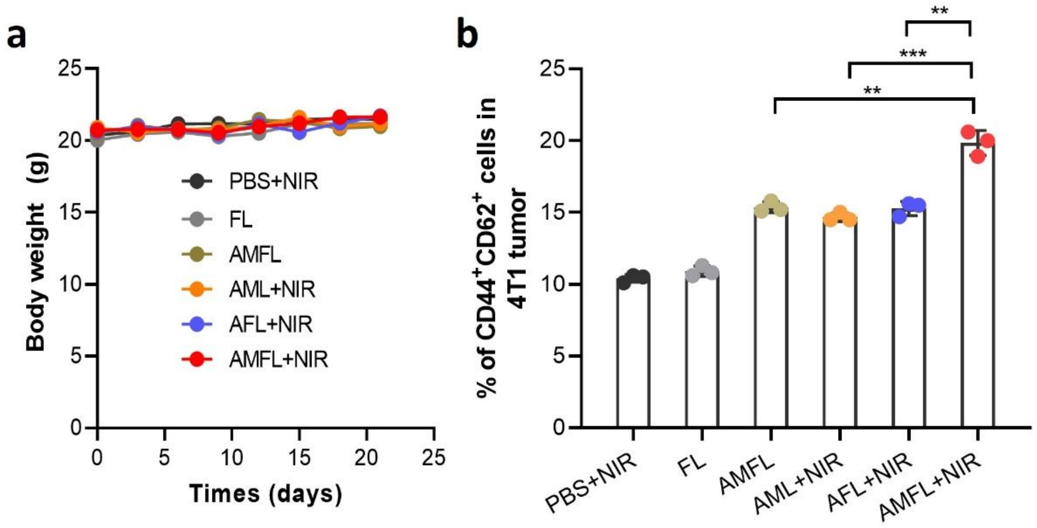

Figure S33. (a) Body weight curves of mice model in Fig. 7 with different treatments. (b) Percentage of central memory T cells ($CD44^{+}CD62^{+}$) in the tumor. (n = 3) Statistical significance was determined using one-way analysis of variance (ANOVA): ***P* < 0.01 and ****P* < 0.001.

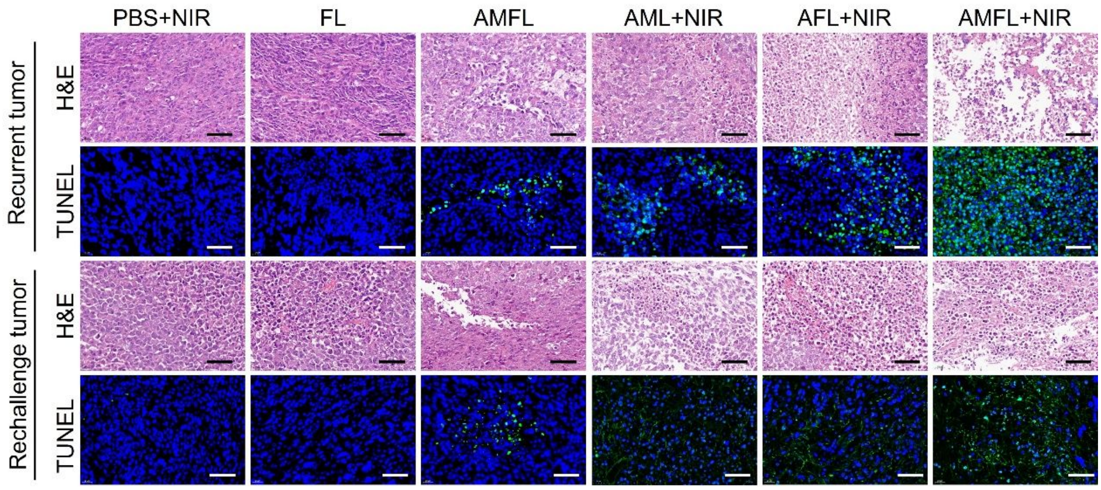

Figure S34. H&E and TUNEL staining of recurrent and rechallenge tumor slices collected after different treatments. Scale bar: 50 μm.

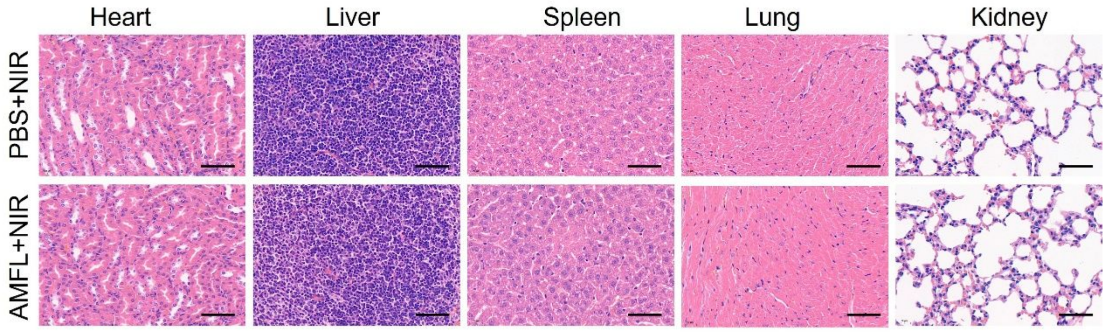

Figure S35. Histological analysis of H&E stained slices of the main organs of mice after different treatments. Scale bars: 50 μm.

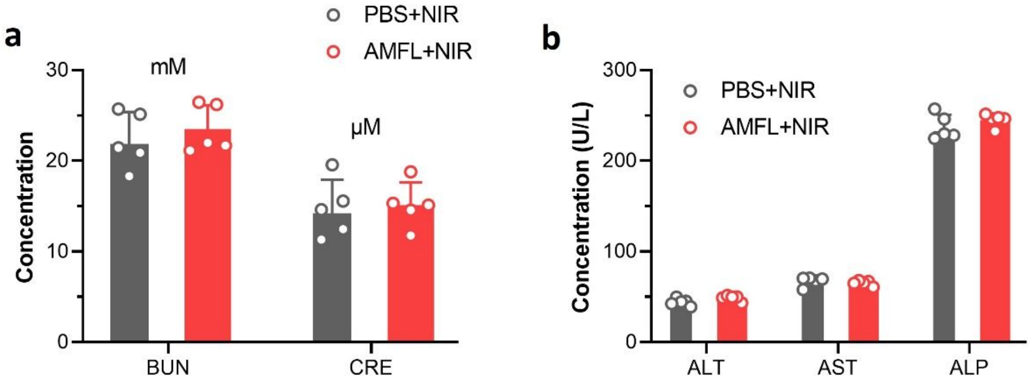

Figure S36. Serum biochemical data of liver and renal function of mice after 21 days of PBS and ATH after injection and therapeutic evaluation. n = 5.

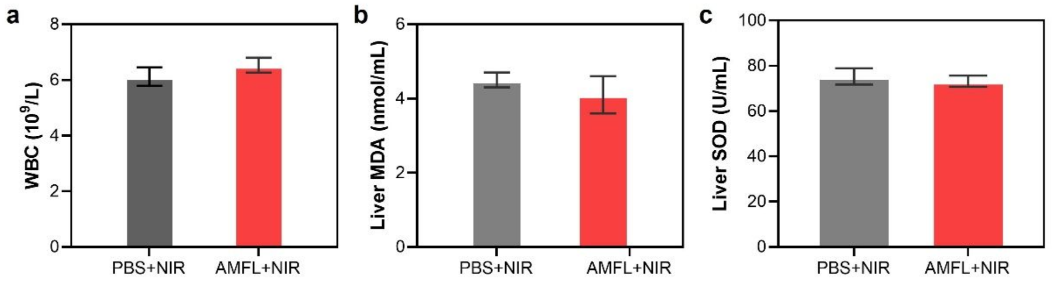

Figure S37. Biochemical assessment for blood Inflammatory indicators (White Blood Cells, WBC) and the liver tissues of the mice after different treatments. Biomarkers include malondialdehyde (MDA) and superoxide dismutase (SOD). n = 3.
